# Construction of a synthetic *Saccharomyces cerevisiae* pan-genome neo-chromosome

**DOI:** 10.1038/s41467-022-31305-4

**Published:** 2022-06-24

**Authors:** Dariusz R. Kutyna, Cristobal A. Onetto, Thomas C. Williams, Hugh D. Goold, Ian T. Paulsen, Isak S. Pretorius, Daniel L. Johnson, Anthony R. Borneman

**Affiliations:** 1 The Australian Wine Research Institute, PO Box 197, Glen Osmond, SA 5064 Australia; 2 ARC Centre of Excellence in Synthetic Biology and Department of Molecular Sciences, Macquarie University, Sydney, NSW 2019 Australia; 3 New South Wales Department of Primary Industries, Elizabeth Macarthur Agricultural Institute, Woodbridge Road, Menangle, NSW 2568 Australia; 4 The Chancellery, Macquarie University, Sydney, NSW 2109 Australia; 5 School of Wine, Food and Agriculture, The University of Adelaide, Adelaide, SA 5005 Australia; 6 Present Address: The Chancellery, Macquarie University, Sydney, NSW 2109 Australia

**Keywords:** Genomic engineering, Synthetic biology

## Abstract

The Synthetic Yeast Genome Project (*Sc*2.0) represents the first foray into eukaryotic genome engineering and a framework for designing and building the next generation of industrial microbes. However, the laboratory strain S288c used lacks many of the genes that provide phenotypic diversity to industrial and environmental isolates. To address this shortcoming, we have designed and constructed a neo-chromosome that contains many of these diverse pan-genomic elements and which is compatible with the *Sc*2.0 design and test framework. The presence of this neo-chromosome provides phenotypic plasticity to the *Sc*2.0 parent strain, including expanding the range of utilizable carbon sources. We also demonstrate that the induction of programmable structural variation (SCRaMbLE) provides genetic diversity on which further adaptive gains could be selected. The presence of this neo-chromosome within the *Sc*2.0 backbone may therefore provide the means to adapt synthetic strains to a wider variety of environments, a process which will be vital to transitioning *Sc*2.0 from the laboratory into industrial applications.

## Abstract (web summary)

The Sc2.0 consortia is reengineering the yeast genome. To expand the Sc2.0 genetic repertoire, the authors build a neo-chromosome comprising variable loci from diverse yeast isolates, providing phenotypic plasticity for use in synthetic backgrounds.

## Introduction

By way of its ease of propagation and well-defined genetics, the yeast *Saccharomyces cerevisiae* represents one of the most intensively studied eukaryotic model organisms and was the first for which a fully characterized genome sequence was available^1^. The International Synthetic Yeast Genome Project (*Sc*2.0) has now also positioned *S. cerevisiae* at the forefront of genome engineering, with this species being the first eukaryote to be synthetically engineered at the whole-chromosome scale^2,3^.

Most studies regarding the biology of *S. cerevisiae*, including the initial genome sequencing^1^ and *Sc*2.0 efforts^2,3^, have focused on the laboratory strain S288c (or derivatives thereof). However, there are hundreds of diverse strains of *S. cerevisiae* and many display distinctive phenotypes that provide selective advantage within specific environmental niches or industries (e.g. fermenting wine, leavening bread or brewing beer)^4^. These phenotypic differences are the direct result of intraspecific genetic variation, often in the form of strain-specific genes or gene clusters^5–11^. The differential presence of these genes between strains can impart striking phenotypic consequences, including the ability to synthesize vitamins or to survive specific environmental stresses or inhibitory compounds^8,12–15^. Interestingly, the common theme across these strain comparisons relative to S288c, is that this laboratory strain appears to represent an almost minimal core of common genes, displaying few open reading frames (ORFs) that are absent in most other strains (except for a large number of transposon integrations), which likely reflects genetic streamlining afforded by selection under ideal laboratory growth conditions^16^. Studies that focus solely on this strain do not therefore consider these pan-genomic ORFs and their phenotypic impacts.

To address this missing genetic variation and provide the potential for additional phenotypic plasticity in the *Sc*2.0 parental strain, we have sought to assemble an array of pan-genomic elements, normally associated with industrial or environmental isolates of *Saccharomyces cerevisiae* into a seventeenth chromosome for inclusion within the *Sc*2.0 background.

## Results and discussion

### Design and de novo synthesis of a pan-genome neo-chromosome

**Fig. 1:**
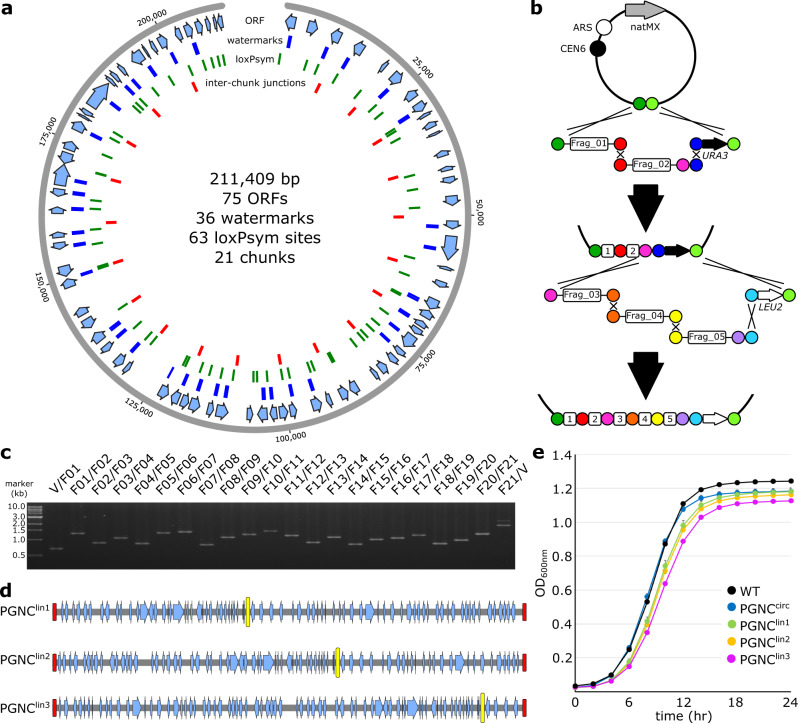
Construction of a yeast pan-genome neo-chromosome. **a** Schematic representation of the circular PGNC construct (PGNC^circ^). **b** Iterative assembly of the PGNC via homologous recombination and using alternating selectable markers. **c** Confirmation of the PGNC assembly using PCR reactions that span each inter-chunk junction. Identical PCR results were obtained across at least two repetitions and during sub-assembly **d** Production of three linear PGNC variants using the telomerator (PGNC^lin1^, PGNC^lin2^ and PGNC^lin3^). Yellow boxes define the position of the centromeric-plasmid backbone. Red boxes denote positions of synthetic telomeric repeat sequences added by the telomerator process. **e** Microplate growth kinetics of the four PGNC variants (PGNC^circ^, PGNC^lin1^, PGNC^lin2^ and PGNC^lin3^) compared to the BY4742 (WT) strain. Data are presented as mean values +/− SD, based upon values obtained from three independent biological replicates. Source data are provided as a Source Data file.

As building blocks for this pan-genome neo-chromosome (PGNC), seventeen unique pan-genome sequences (1.1–60.3 kb), were identified from across whole-genome sequences of more than 200 diverse strains of *S. cerevisiae*^16^. The final collection comprised a non-degenerate set of sequences from eight wine, sake, biofuel, human pathogen and natural isolates (Supplemental Dataset 1). These fragments were concatenated in silico into a single DNA molecule, to which global systematic changes were introduced in accordance with the *Sc*2.0 project^2^. This included the substitution of TAA for TAG stop-codons, the introduction of oligonucleotide watermarks within 36 ORFs (Supplementary Table 1, Supplementary Fig. 1) and 63 bi-directional Cre-recombinase recognition (loxPsym) sites. The total length of the final synthetic PGNC was 211,409 bp and contained 75 predicted ORFs (Fig. 1a). The sequences of each ORF, along with strain origins and functional annotation are provided in Supplementary Dataset 1, with a full annotated sequence of the PGNC presented in Supplementary Dataset 2.

To allow for DNA synthesis, the final PGNC design was divided into 21 fragments (chunks), of ~10 kb in length (Supplementary Fig. 1). Each chunk was flanked by 200 bp of overhanging sequences at both termini, which were designed to allow for in vivo assembly in *S. cerevisiae* via homologous recombination. Two auxotrophic marker genes, *URA3* and *LEU2*, were also synthesized with specific flanking sequences, allowing for them to be alternatively integrated during the processive steps of the assembly (Fig. 1b).

A yeast centromeric vector (p416-natR) was used as the backbone for the neo-chromosome assembly, which provided a functional centromere fused to an autonomously replicating sequence and a nourseothricin (clonNAT) resistance marker (Fig. 1b). Assembly was initiated with linearized p416-natR, two pan-genome chunks (Frag_01, Frag_02) and the *URA3* marker. For the second round of assembly three chunks (Frag_03, Frag_04, Frag_05) were introduced into a strain containing the completed first round assembly, along with the alternative auxotrophic marker (*LEU2*). Eight rounds of assembly were ultimately conducted, with between one and four pan-genome chunks incorporated per cycle. A ninth round was then used to replace the remaining *LEU2* marker with a BFP-expression cassette, producing the final, circular neo-chromosome (PGNC^circ^). The integrity of the PGNC^circ^ strain was then confirmed by both PCR across each inter-chunk junction (Fig. 1c, Supplementary Fig. 2) and by whole-genome sequencing.

*S. cerevisiae* has been shown previously to be able to host large heterologous episomes, such as whole bacterial genomes, in yeast-bacterial shuttle vectors^17,18^. Circular variants of native *S. cerevisiae* chromosomes have also been engineered as part of the *Sc*2.0 consortium, where they behave normally, except during meiosis^2,19^. To compare the behaviour of circular and linear chromosomal variants of the PGNC, linearized versions were engineered using the telomerator^20^ at three different loci within PGNC^circ^ (Fig. 1d, Supplementary Fig. 2). This resulted in three linear chromosomal variants (PGNC^lin1^, PGNC^lin2^ and PGNC^lin3^), which differed only in the arrangement of genes relative to the newly introduced telomeric sequences. Growth curves were performed to assess the effect of these PGNC chromosomal variants on overall strain fitness in rich media (Fig. 1e). While PGNC^circ^ displayed a growth curve that was comparable to the wildtype strain, the linear variants all displayed slightly extended lag periods and reduced total cell densities and maximum specific growth rates (WT, 0.53 h^−1^; PGNC^circ^, 0.53 h^−1^; PGNC^lin1^, 0.51 h^−1^; PGNC^lin2^, 0.5 h^−1^ and PGNC^lin3^, 0.46 h^−1^). All the PGNC variants displayed a lower final optical density than the parental strain, indicating that PGNC elements were impacting strain fitness under standard laboratory conditions.

### PGNC stability

**Fig. 2:**
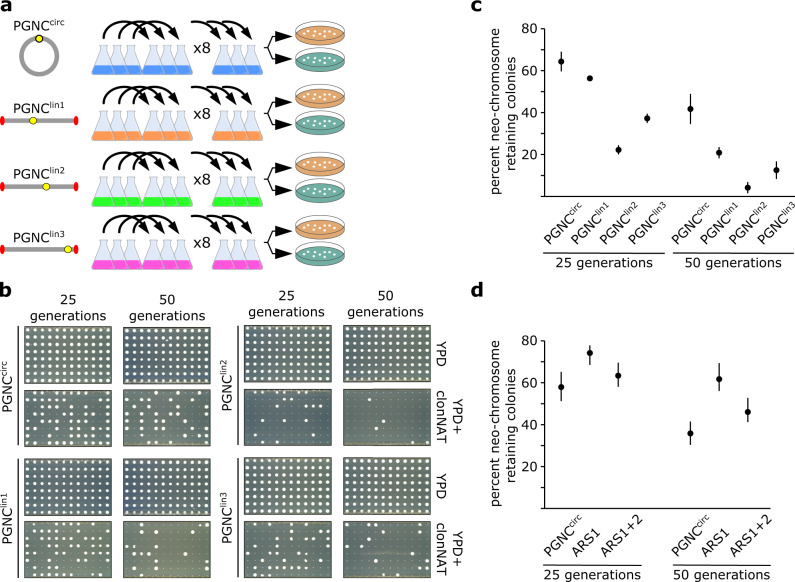
Mitotic stability of the PGNC in the absence of selection. **a** Populations were established for each four PGNC variants (PGNC^circ^, PGNC^lin1^, PGNC^lin2^ and PGNC^lin3^) and serially passaged eight times for ~50 generations of total growth. **b** Differential plating of 96 colonies for phenotypic assessment of loss of the PGNC element. **c** Results of passaging experiments (96 colonies per replicate) for each of the PGNC strains after both 25 and 50 generations. **d** Results of passaging experiments (96 colonies per replicate) for each of the PGNC strains containing additional ARS sequences after both 25 and 50 generations. All graphs are presented as mean values +/− SD, based upon values obtained from three independent biological replicate passaging experiments. Source data are provided as a Source Data file.

PGNC^circ^ is only 20 kb smaller than the native chromosome I of *S. cerevisiae* (smallest native chromosome) and growth curves suggested that it may impart a selective disadvantage to the PGNC-carrying strains in the absence of clonNAT-induced selection. To assess the mitotic stability of the circular and linear variants of the PGNC, representative strains were serially passaged under non-selective (media without clonNAT) conditions. Isolates from each population were assessed for the presence of the PGNC after 25 and 50 generations (Fig. 2, Supplementary Figs. 4 and 5). PGNC^circ^ displayed the highest stability, averaging 61.7 ± 4.5% and 40 ± 6.9% retention over 25 and 50 generations, respectively. Of the linear versions, PGNC^lin1^ displayed the highest retention (25 gen, 54.0 ± 1.0%; 50 gen, 20.0 ± 2.6%), while PGNC^lin2^ was very unstable, with only 21.3 ± 2.1% (25) and 4.0 ± 2.6% (50 generations).

To attempt to address the stability issues of the PGNC, additional ARS sequences were inserted into the PGNC at two different sites. Two variants were made to the PGNC^circ^, whereby either a single copy of ARS305^21^ (efficient, early firing origin from chromosome III) was inserted between ORF58 and ORF59 (ARS_305_01), or a dual-variant with a second ARS305 inserted between ORF19 and ORF20 (ARS_305_02) (Supplementary Fig. 1). The inclusion of the additional ARS sequences were shown to modestly improve the stability of the PGNC^circ^ element, although not to levels that would preclude the use of selective media for the long-term stability of the PGNC (Fig. 2d). Complicating these results, the dual-variant did not provide increased stability relative to the ARS_305_01 alone, suggesting that there is a multi-factored interplay between total ARS number and overall stability.

In future studies, improvement in the stability of the PGNC element could be investigated through screening of additional combinations of positions of ARS elements or through the addition of an essential gene or fusion to another chromosome, as reported for synI^22^, to drive the maintenance of this element without requiring drug-based selection.

### PGNC imparts distinct phenotypes in the Sc2.0 strain background

**Fig. 3:**
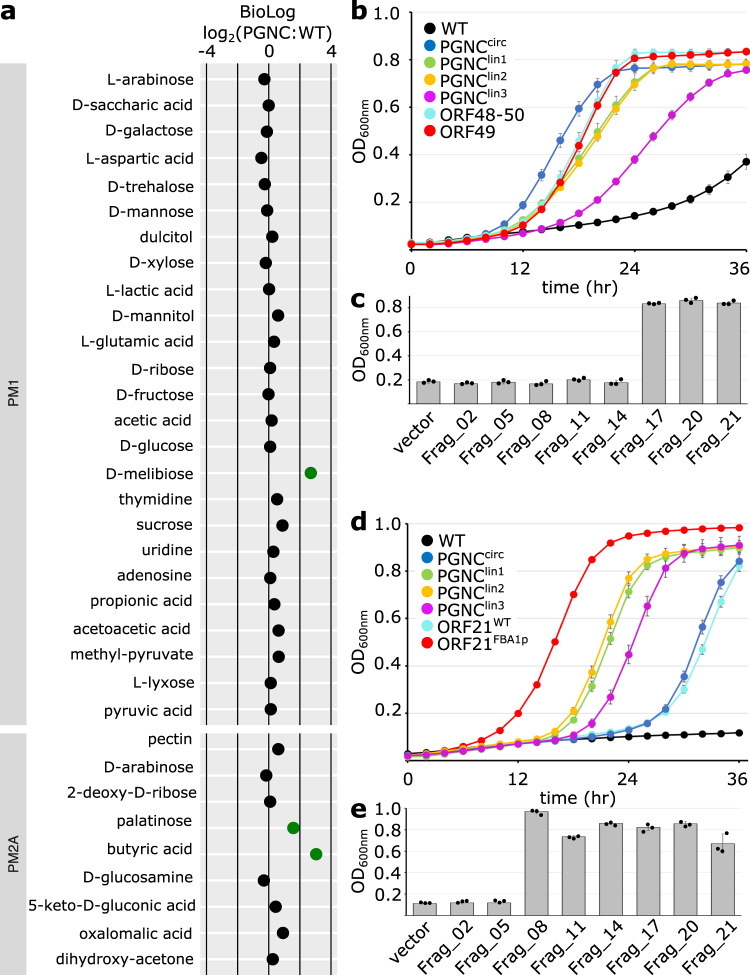
The PGNC element expands the phenotypic repertoire of BY4742. **a** BioLog results for the carbon-source utilization plates, PM1 and PM2A. Values are presented as log_2_ normalized (PGNC:WT) observations of maximal respiration rate, with increased growth rates highlighted in green. **b** Growth kinetics for PGNC strains with palatinose as a sole carbon source. **c** Identifying the palatinose-utilizing region of the PGNC via 24 h end-point growth assays of intermediate PGNC elements. **d** Growth kinetics for PGNC strains with melibiose as a sole carbon source. **e** Identifying the melibiose-utilizing region of the PGNC via 24 h end-point growth assays of intermediate PGNC elements. All growth kinetic data are presented as mean values +/− SD, based upon values obtained from three independent biological replicates. Source data are provided as a Source Data file.

Given the coding potential of the PGNC, in addition to existing reports of phenotypic outcomes of some of the genes known to be present within the neo-chromosome^12,23^, the phenotypic consequence of the presence of PGNC in BY4742 was compared to the parent strain using the BioLog Phenotype Microarray^24^ (Supplemental Dataset 3). Analysis of the BioLog results demonstrated nine conditions in which PGNC led to at least a two-fold increase in BioLog output (maximum curve height) compared to the BY4742 parent (D-melibiose, palatinose, butyric acid, 5% sodium formate, 4% sodium lactate, neomycin, FCCP, deoxy-D-glucose and ibuprofen) and eight conditions in which relative growth was reduced more than two-fold in the PGNC strain (benserazide, magnesium chloride, caffeine, EGTA, isoniazid, methyl-viologen, tamoxifen and microazole nitrate) (Supplemental Dataset 3). Differential carbon source utilization provided the clearest examples of selective growth of the PGNC strain, with melibiose, palatinose and butyric acid being utilized only in the presence of PGNC (Fig. 3a). As the utilization of the carbon sources, palatinose and melibiose were expected to be due to the presence of specific glycosidases^25–28^ (with potential candidate enzymes annotated in the PGNC), these compounds were selected for additional, larger-scale fermentation to confirm the BioLog results and map the regions of the PGNC that were responsible.

Palatinose (Isomaltulose) is a disaccharide composed of glucose and fructose linked via an alpha-1,6-glycosidic bond. While *S. cerevisiae* BY4742 encodes several isomaltases^25,26^, this strain shows very slow utilization of palatinose (Fig. 3b). In contrast, PGNC strains show efficient palatinose utilization, reaching a stationary phase within 24 h (Fig. 3b). To define the pan-genome ORF(s) that might be responsible for this phenotype, intermediate PGNC strains (produced during the stepwise assembly process) were tested for the palatinose-utilization phenotype (Fig. 3c). While strains containing the chunks Frag_01 through Frag_14 displayed a non-utilizing phenotype, intermediate strains containing Frag_15 to Frag_17 displayed a phenotype that was indistinguishable from the full PGNC. Functional annotation of ORFs within Frag_15 provided a candidate cluster of three ORFs, predicted to encode an alpha-1,6-glycosidase family enzyme (ORF48), a putative zinc-finger transcription factor (ORF49) and a sugar transporter (ORF50) (Supplementary Fig. 1). The presence of this cluster of ORFs alone (ORF48–50) was shown to also provide robust growth on palatinose (Fig. 3b). When the function of this cluster was further refined by the expression of individual ORFs, ORF49 was shown to provide the same levels of growth as the three ORF clusters. The putative transcription factor encoded by ORF49 is therefore responsible for the palatinose-utilisation phenotype and the ability for this ORF to stimulate utilisation of palatinose in the S288c background, presumably occurs through activation of MAL-family glucosidases that are present in the S288c genome^27^.

Melibiose is a disaccharide composed of galactose and glucose, which are linked via an alpha-1,6-glycosidic bond. Unlike palatinose, *S. cerevisiae* BY4742 is unable to utilize melibiose as a sole carbon source, even under extended periods of growth^28^, while all of the PGNC variants displayed robust growth on this sugar (Fig. 3d). Analysis of the PGNC intermediates located the region responsible for this phenotype within Frag_06 to Frag_08, which contained 11 predicted ORFs (Fig. 3e). From this group, ORF21, predicted to encode an α-galactosidase, was the clear candidate for this phenotype. Expression of ORF21 in isolation was subsequently shown to be sufficient for the melibiose-utilizing phenotype and the over-expression strain (ORF21^FBA1p^) displayed significant enhancement in its utilization of melibiose (Fig. 3d), confirming the role of this ORF.

### SCRaMbLE-induced phenotypic diversity

**Fig. 4:**
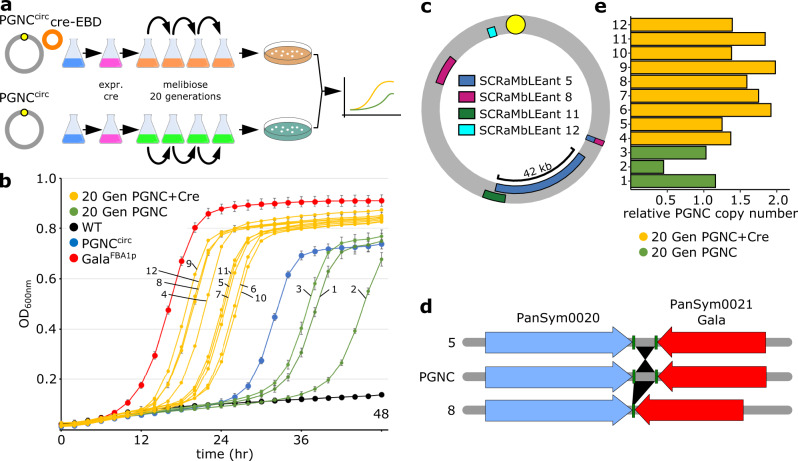
SCRaMbLE induced genetic and phenotypic diversification of the PGNC. **a** The methodology for SCRaMbLE-induced selection for increased growth on melibiose. **b** Growth kinetics of individual isolates from Cre-expressing and control populations. Data are presented as mean values +/− SD, based upon values obtained from three independent biological replicates. **c** Locations of clone-specific SCRaMbLE-induced recombination between loxPsym sites within the PGNC element. **d** Structural variation adjacent to ORF21. **e** SCRaMbLE-induced copy number variation within the melibiose-selected isolates (yellow, isolates from the Cre-expressing populations; green, isolates from the control populations). Source data are provided as a Source Data file.

One of the key attributes of the *Sc*2.0 design is the ability to stimulate genetic diversity through recombination-mediated rearrangement of the loxPsym sites (termed SCRaMbLE) that were inserted throughout the *Sc*2.0 synthetic chromosomes^2,29^. As the PGNC design included 63 loxPsym sites, the effect of SCRaMbLE on the structure of the PGNC was investigated. Given the clear melibiose-utilization phenotype provided by the PGNC, combined with the evidence for further improvement in growth (provided by the ORF21^FBA1p^ results), this phenotype was chosen as a test for SCRaMbLE-induced adaptive improvement. As the PGNC^circ^ element displayed growth kinetics closest to the parental strains (Fig. 1e), the strain containing this element was chosen as the basis for the adaptive experiments. SCRaMbLE was performed on the strain containing the PGNC^circ^ element, with the resulting mixed population subjected to competitive growth on melibiose (Fig. 4a). After serial passaging, nine single colonies from the Cre-expressing population (Cre^+^) and three colonies from the control population (Cre^-^) were assessed for growth of melibiose relative to the PGNC^circ^ and ORF21^FBA1p^ strains (Fig. 4b). Of the twelve isolates, all nine from the Cre^+^ population displayed growth rates on melibiose that were significantly improved relative to PGNC^circ^, although none were able to match the very high growth rate that was observed with ORF21^FBA1^ (Fig. 4b). The three isolates that were selected from the Cre^-^ population did not show an adaptive response to melibiose and displayed lower growth rates in response to the extensive passaging during the SCRaMbLE procedure. In addition, growth rates varied substantially between individual isolates, suggesting that the SCRaMbLE process was producing genetic diversity as expected.

To directly observe the genetic response that accompanied the SCRaMbLE induction and melibiose selection, all twelve phenotyped isolates were subjected to nanopore-based whole-genome sequencing. No structural variation was observed in the control samples; however, four of the strains from the Cre^+^ population displayed structural variations consistent with recombination between loxPsym sites (Fig. 4c, Supplementary Table 2). Two strains displayed structural rearrangements that were in the intergenic region at both the 3′ end of ORF21 and the adjacent ORF (ORF20), with strain 5 displaying an inversion of the intergenic regions and strain 8 having a deletion of this same region (Fig. 4d). In addition to structural variation, the whole-genome sequencing afforded the ability to investigate the copy-number variation of the PGNC element (Fig. 4e), where, compared to the control isolates, the Cre^+^ population displayed a significantly increased relative copy number of the entire element (*p* = 0.0042). It is unclear how the expression of the Cre-recombinase led to these changes in relative copy-number; however, as both the Cre^+^ and control populations were selected on melibiose, this effect does appear to be due to the expression of the recombinase.

At this stage, it is not known how these combined structural variants influenced the ability of these strains to utilise melibiose, especially given that strains contained multiple individual mutations that may be synergistic, additive or neutral. While it is relatively straightforward to reconcile increased PGNC copy-number with increased expression of ORF21 (and melibiose utilisation), hypotheses pertaining to other variants are harder to postulate, although the alterations to the 3′ untranslated region of ORF21 could suggest that altered transcript stability may be responsible for some increases that were observed. The processes underpinning the increased growth rate of the two SCRaMbLEd strains with no detectable structural variation or increased copy number (isolates 4 and 10 in Fig. 4) may be due to alterations in the interplay between the natural genome and the PGNC or to smaller-scale mutations such as single-nucleotide polymorphisms.

In summary, the *Sc*2.0 chassis provides a framework for engineering the next era of industrial microbes. The ability to introduce neo-chromosomes, such as the PGNC, has been demonstrated to greatly expand the genetic and phenotypic diversity that can be achieved within the *Sc*2.0 background. This provides the means to adapt this, and other, synthetic strains to a variety of environments, a process which will be vital to transitioning *Sc*2.0 from the laboratory into industrial applications.

## Methods

### PGNC design

A total of 17 unique pan-genome sequences (1.1–60.3 kb), were selected from whole-genome sequences of more than 200 diverse strains of *S. cerevisiae*^9^. These fragments were concatenated in silico in descending size order into a single DNA molecule, to which global systematic changes were introduced in accordance with the *Sc*2.0 project^2^. In short, these changes included: the substitution of TAA stop-codons for TAG stop codons, the introduction of oligonucleotide watermarks in 36 ORFs using the principles of codon redundancy (Supplementary Table 1) and the introduction of 63 bi-directional Cre-recombinase recognition sequences (loxPsym), located 3 bp after the stop codon of selected high-confidence ORFs.

Functional annotation for the 75 predicted ORFs (Supplemental Dataset 2) was performed using the Interproscan 5 pipeline v. 5.52–86.0^30^. Annotation of Carbohydrate-active enzyme (CAZYme) classes was performed through an HMMer search v.3.3.2^31^ of the dbCAN HMMdb v.9.0 database^32^. KEGG orthology assignments were obtained using BlastKOALA v.2.2^33^ and prediction of signal peptides was performed using SignalP v.4.1^34^.

For in vivo assembly, the PGNC was divided into 22 fragments (chunks) of ~10 kb in length (Supplementary Fig. 1). Each chunk was flanked with *Pme*I and/or *Not*I restriction sites to allow for release from the plasmid vector backbone (pUG57), in addition to 200 bp of overhanging sequences at the 5′- and 3′-termini, which were homologous to their neighbouring fragments. Two auxotrophic markers, *URA3* and *LEU2*, were also designed with specific flanking sequences, allowing them to be alternatively integrated during the processive steps of the assembly.

### PGNC synthesis and assembly

All 21 chunks and the selectable markers cassettes were synthesized and cloned into a plasmid vector (GenScript). For the neo-chromosome assembly, the yeast centromeric vector p416-natR created by replacing *URA3* auxotrophic marker with clonNAT resistance marker in p416-GPD vector^35^ (Supplementary Fig. 1). Assembly was initiated with linearization of the p416-natR vector with *Cai*I endonuclease and release of Frag_01, Frag_02 and *URA3* from pUG57 using *Pme*I and *Not*I. Fragments were pooled in even ratios and transformed into *S. cerevisiae* (BY4742). After transformation, cells were selected on a solid yeast nitrogen base (YNB) medium lacking uracil and supplemented with 100 µg/mL of clonNAT (YNB-Ura+clonNAT) and incubated at 30 °C for 72 h. Colonies were confirmed using PCR, with confirmation primers designed to amplify across the junctions between each pair of adjacent chunks (Supplementary Table 3).

For the second round of assembly, the chunks Frag_03, Frag_04, Frag_05 were introduced into a strain containing the confirmed first-round assembly product, along with the alternative auxotrophic marker (*LEU2*). In total, 7 rounds of assembly were conducted, with a varying number of assembled synthetic pan-genomic DNA molecules (1–4 per assembly cycle), while alternating the auxotrophic markers. The complete set of diagnostic PCRs, utilizing the primer combinations from Supplementary Table 3, were conducted on the final strain carrying the completed PGNC.

The remaining *LEU2* auxotrophic marker, which was present in the sequence of PGNC after the last round of the assembly, was removed using selection/counter selection approach using the CORE7 cassette^36^.

For the selection step, the CORE7 cassette was PCR amplified from the plasmid using primers equipped with 50 bp flanking regions that were homologous to sequences directly flanking the *LEU2* gene (Supplementary Table 4). The CORE7 cassette was transformed into yeast and selected on solid YPD medium supplemented with 100 µg/mL of Hygromycin B (Sigma-Aldrich). Transformant colonies were tested for successful CORE7 cassette insertion using PCR with the primers FR21-F and FR2-p416-R (Supplementary Table 3).

A single transformant that displayed the expected PCR pattern was then used for the counter-selection step. Here, the *FBA1*_*p*_::*BFP2*::*PGK1*_t_ cassette, which was amplified from pCV2 vector using primers equipped with 50 bp of homologous sequences up- and down-stream of the inserted CORE7 cassette (Supplementary Table 3). This was transformed into yeast, with cells plated onto solid YNB media, supplemented with 20 g/L galactose as a sole carbon source (YNB-Gal). Transformant colonies were screened for the successful removal of CORE7 using PCR (primers FR21/V-F and FR21/V-R, Supplementary Table 1).

### Removal of auxotrophic markers mutations

The *S. cerevisiae* BY4742 strain carries four separate auxotrophic mutations: *his3*∆1, *leu2*∆0, *lys2*∆0 and *ura3*∆0^37^. These mutations were cured from the parent strain by replacing each mutated locus with functional sequences that were PCR amplified using genomic DNA of a prototrophic strain. *HIS3*, *LEU2* and *LYS2* PCR products were pooled in equal amounts and transformed into BY4742 containing PGNC^circ^ element. Transformants were selected on solid YNB-clonNAT medium lacking histidine, leucine and lysine. Transformant colonies were tested for correct genomic integration using PCR (Supplementary Table 5). The *URA3* auxotrophic mutation was not addressed at this stage as *URA3* auxotrophy was needed as a marker for the introduction of the telomerator (see below).

### Construction of a *Sce*I expression vector

A *Sce*I expression vector (pTL85-SceI), was constructed from two yeast shuttle vectors, pTL85^38^ (Dr. Tiziana Lodi, University of Parma, Italy) and pUDC073^39^ (Euroscarf). Both vectors were digested with *Pvu*II, in case of pTL85, this resulted in the isolation of the plasmid backbone, which carried the kanamycin resistance cassette (KanMX), and in the case of pUDC073, a partial *Pvu*II digestion resulted in the isolation of the *GAL1*_p_::*Sce*I::*CYC1*_t_ cassette (Supplementary Fig. 9). All restriction fragments were purified from the agarose gel (Wizard SV Gel and PCR Clean-Up System, Promega) prior to ligation (Blunt/TA Ligase Master Mix, New England BioLabs). Ligations were transformed into high efficiency NEB 10-beta Competent *E. coli* (New England BioLabs) following the manufacturer’s instruction and confirmed by restriction digest.

### Linearization of the PGNC

The PGNC^circ^ element was linearized using the telomerator^20^ (Supplementary Fig. 2). The telomerator cassette was synthesized (Genescript) and PCR amplified from the vector using primers equipped with 50 bp long flanking regions, homologous to one of three genomic locations within the PGNC (Supplementary Fig. 2a, Supplementary Table 6).

The PGNC^circ^ strain was transformed with each of the three separate telomerator PCR products, to insert the cassette in three distinct locations (PGNC^lin1^, PGNC^lin2^ and PGNC^lin3^) (Supplementary Fig. 2a). Transformed strains were selected on solid YNB -Ura +clonNAT medium. Insertion at the expected location was tested by PCR (Supplementary Table 7). Strains with correct telomerator insertions were then tested for lack of growth on agar plates containing 1 µg/mL of 5-fluoroorotic acid (5-FOA), which selects for loss of the *URA3* marker. To confirm the purity of the selected telomerator variants, strains were plated out in serial dilutions onto YPD rich medium containing 100 µg/mL clonNAT and incubated for 48 h at 30 °C along with the control strain (PGNC^circ^). Isolates of each variant were then transferred onto YNB, YNB - URA, and YNB + 1 mg/mL of 5-FOA, all containing 100 µg/mL clonNAT.

To induce linearization of the telomerator, confirmed strains were transformed with the pTL85-SceI vector. Transformed strains were plated onto solid YNB medium supplemented with 100 µg/mL of clonNAT (selecting for the PGNC) and 200 µg/L G418 (selecting for pTL85-SceI). Transformant colonies were tested for the presence of pTL85-SceI vector using PCR with M13 primers.

Three transformants (each carrying the telomerator in distinct location), as well as the control strain carrying only the PGNC were then inoculated into separate YPD cultures supplemented with 100 µg/mL of clonNAT, 200 µg/L G418, and incubated overnight, with shaking, at 30 °C. Cells from these cultures were harvested by centrifugation and washed twice with dH_2_O and inoculated (OD_600_ 0.1) into YPGal (YPD with 10 g/L galactose as a sole carbon source) medium, supplemented with 100 µg/mL of clonNAT and 200 µg/L G418 and incubated for 24 h at 30 °C. After incubation in YPGal medium, cultures were washed in dH_2_O and serial dilutions were plated onto solid YNB media containing 1 µg/mL of 5-FOA and 100 µg/mL of clonNAT and incubated for 72 h at 30 °C. Transformant colonies were tested for the linearization by the telomerator using PCR primers specific to each of the three distinct regions where the telomerator was to be inserted (Supplementary Fig. 2c, Supplementary Table 7).

Following linearization, the pTL85-SceI vector was removed by growth under non-selective conditions for the plasmid (5–6 generations). To phenotypically test successful linearization, ten colonies of each strain were then pinned onto YNB, YNB -URA, and YNB + 5-FOA (all containing 100 µg/mL clonNAT), (Supplementary Fig. 3). After the completion of the linearization process all stains were cured of the *URA3*∆0 mutation using transformation-based method described above.

### Mitotic stability of the PGNC variants

The mitotic stability of the circular and three linearized variants of the PGNC were tested using replicative colony picking. Strains were grown overnight in YPD supplemented with 100 µg/mL of clonNAT. YPD cultures (100 mL) were then inoculated in triplicate (OD_600_ 0.1) and incubated at 30 °C for 24 h before being diluted and passaged into fresh YPD medium (OD_600_ 0.1). Passaging was repeated ten times (~50 generations of non-selective growth). Single colonies per replicate were pinned onto both solid YPD medium and YPD + clonNAT medium with a PIXL robotic system (Singer Instruments) and incubated at 30 °C for ~72 h, with the proportion of clonNAT resistant and sensitive colonies used to infer stability.

Autonomously Replicating Sequence (ARS 305) was introduced to the PGNC using CRISPR/Cas9 methodology. pCAS plasmid (ATUM) expressing Cas9 endonuclease under control of *RNR2* promoter, single guide RNA (sgRNA) sequence and kanMX selection was used for yeast transformations. The sgRNA sequences (20-mer protospacer) were designed using CRISPR gRNA Design tool (ATUM). Confirmed pCAS vectors were transformed into yeast along with the DNA fragments containing ARS305 sequence and 200 bp of flanking sequence homologous to the intended ARS insertions sites on the PGNC.

### BioLog phenotyping

Analysis of growth in the presence of an array of different nitrogen sources, carbon sources, and potentially toxic compounds was assessed using a BioLog Phenotype Microarray^24^ with the growth of PGNC^lin1^ compared with BY4742 containing the pFA-TagRFP-T-CdHIS1 plasmid. Plates PM1 and PM2 were supplemented with dye mix D. Plates PM3B, PM4A, PM5, PM6, PM7, and PM8 were supplemented with 100 mM D-Glucose and dye mix D. Plates PM9, PM10, PM20B, PM21D, PM22D, PM23A, PM24C, and PM25D were supplemented with 100 mM D-Glucose and dye mix E. Plates were incubated at 30 °C and sampled every 15 min for 24 h. References of the location on the Microplate to the compounds tested could be found on the manufacturer's website (www.biolog.com/products-portfolio-overview/phenotype-microarrays-for-microbial-cells). Plates, 6x concentrated dye mixes, turbidimeter and media were sourced from BioLog (Hayward, CA 94545 USA).

Raw BioLog data were analyzed using the R package opm^40^ to extract values for maximum curve height (A). Data were then exported and log_2_ ratios calculated for pairs of values from the PGNC^lin1^ and WT plates. Compounds producing log_2_ ratios of PGNC:WT maximum curve heights ≥1 were classified as displaying an increased growth rate for that compound. Wells were excluded from analysis if at least one strain did not exceed the negative control value by at least 50 units or if the positive control for the plate failed to reach 100 units.

### Constructing α-galactosidase expressing vectors

p416-natR-Gala was constructed utilizing the p416-natR backbone. The Gala gene sequence (ORF with 795 bp upstream and 291 bp downstream), was PCR amplified (KAPA2G Robust PCR Kit, Sigma-Aldrich), using primers containing *Xho*I restriction at the 5′ end. The PCR product and p416-natR vector were then digested (*Xho*I) and ligated.

To create Gala^FBA1p^, the *FBA1* promoter was PCR amplified (KAPPA, Sigma-Aldrich) using primers containing either *Not*I or *Spe*I restriction sites at −5′ ends. The α-galactosidase ORF and 215 bp of its terminator region were PCR amplified with primers containing either *Spe*I or *Xho*I restriction sites. The p416-natR vector was digested with *Not*I and *Xho*I and dephosphorylated using calf intestinal alkaline phosphatase CIP (New England BioLabs).

### Microplate growth assays

Pre-inoculum cultures were established from initial YPD cultures (OD_600_ of 0.1) and incubated for 16–18 h. Cells from the pre-inoculum cultures were then harvested by centrifugation, washed twice in the experimental medium and then diluted to OD_600_ ~ 0.025. 200 µL aliquots were then dispensed in triplicate to random wells of a 96-well flat-bottomed microtiter plate. Microtiter plates were sealed using gas-permeable membranes (Breathe-Easy) and incubated at 30 °C. The growth of cultures was monitored by absorbance (OD_600_) using a TECAN Infinity 200 plate reader. Specific growth rate (*U*) was determined by

1 $$U=\frac{({{{{{\rm{ln}}}}}}(x/{x}_{o}))}{t}$$

where *x* and *x*_o_ are the observed OD_600_ values and *t* is the time (hours) between the observations.

### Construction of pTL85-Cre-EBD and pTL85-Ctrl vectors

pTL85-Cre-EBD was constructed by combining two shuttle vectors, pTL85^38^ and pSH62-EBD^41^ (Addgene). Both vectors were digested with *Pvu*II. For the pTL85 vector, digestion with *Pvu*II provided the plasmid backbone, which carries a kanamycin resistance cassette. For pSH62-EBD, *Pvu*II digestion provided a cassette containing the *GAL1* promoter, Cre recombinase fused to the oestrogen nuclear receptor alpha ligand-binding domain (ER-LBD) and *CYC1* terminator (*GAL1*_*p*_::ER-LBD::*CYC1*_*t*_). Both fragments were gel-purified (Wizard SV Gel and PCR Clean-Up System, Promega) and ligated (Blunt/TA Ligase Master Mix, New England BioLabs). Ligations were transformed into competent *E. coli* cells (NEB 10-beta, New England BioLabs). The control vector, which lacks the *GAL1*_p_::ER-LBD::*CYC1*_t_ element, was created by re-ligating the digested and purified pTL85 vector backbone.

### SCRaMbLE for population-based improvement of melibiose utilisation

Yeast cells carrying the circular version PGNC were transformed separately with the pTL85-Cre-EBD and pTL85-Ctrl vectors. Transformed cells were plated on YPD medium containing clonNAT (to select for the presence of the PGNC), and G418 (to select for the presence of the plasmids). After incubation at 30 °C for 48 h, isolated colonies were inoculated into 50 mL Falcon tubes containing 10 mL of YPD and incubated overnight with shaking at 30 °C. Cultures were harvested by centrifugation, washed once in dH_2_O, and diluted to an OD_600_ of 0.1 in YPGalactose containing 1 μM estradiol (Sigma-Aldrich). Cultures were incubated overnight with shaking at 30 °C, washed twice in dH_2_O and inoculated at an OD_600_ of 0.1 into 20 mL YP-CG (YPD with glucose substituted for 10 % melibiose as a sole carbon source). Incubations were carried out at 30 °C with shaking for 48 h and then passaged back into new media (OD_600_ 0.1). This cycle was repeated four times (~20 generations).

### Genome sequencing and structural variation analyses of the PGNC

Yeast DNA was isolated by lysis of protoplasts formed through zymolyase digestion and potassium acetate precipitation^42^. Sequencing libraries for nanopore whole-genome sequencing were prepared using the Native Barcoding Kit 1D (EXP-NBD104) in combination with the Ligation Sequencing Kit (SQK-LSK109) and loaded into a FLO-MIN106 R9 flow cell. Sequencing was performed using the MinKnow (v19.10.1) on the MinION platform (Oxford Nanopore Technologies, UK).

Fast5 files were base called and demultiplexed using Guppy v.3.2.1 (Oxford Nanopore Technologies, UK). Reads with a minimum qscore of 7 were retained for genome assembly using Canu v.1.7.1^43^, with assemblies polished using Nanopolish v.0.11.2. Contigs that contained the completely resolved PGNC were located by mapping the p416-natR backbone region to the genome assemblies. For structural variation analyses, the PGNC contig was replaced by the original sequence of the PGNC and reads were mapped back to each genome assembly using Minimap2 v.2.17^44^. Structural variants were identified using Sniffles v.1.0.11^45^ and confirmed by manual inspection. The relative copy number of the PGNC was calculated by mapping reads back to each genome assembly using Minimap2 v.2.17^44^ and the ratio between the average coverage of all contigs larger than 200 kb and the PGNC was obtained using CoverM v.0.4.0 (https://github.com/wwood/CoverM).

### Reporting summary

Further information on research design is available in the Nature Research Reporting Summary linked to this article.

## Supplementary information

Supplementary Information

Reporting Summary

Description of Additional Supplementary Files

Dataset 1

Dataset 2

Dataset 3

## Data Availability

The strains and plasmids generated in this study have been deposited and are available from the Australian Wine Research Institute Culture Collection. The raw DNA sequence data generated in this study have been deposited under BioProject accession code PRJNA615683. All other data generated in this study are provided in the Supplementary Information/Source Data files. Source data are provided with this paper.

## Source data

Source Data
