# Supplementary material for: Construction of a synthetic Saccharomyces cerevisiae pan-genome neo-chromosome
Source: Nat Commun. 2022 Jun 24;13:3628. doi: 10.1038/s41467-022-31305-4 (PMC9232646; doi:10.1038/s41467-022-31305-4)
Supplement: Supplementary file 1 — Supplementary Information [file 41467_2022_31305_MOESM1_ESM.pdf]

Supplementary Information

Construction of a synthetic *Saccharomyces cerevisiae* pan-genome neo-chromosome

Dariusz R. Kutyna[1], Cristobal A. Onetto[1], Thomas C. Williams[2], Hugh D. Goold[2,3], Ian Paulsen[2], Isak S. Pretorius[2,4], Daniel L. Johnson[1,5] and Anthony R. Borneman[1,6*]

[1]The Australian Wine Research Institute. PO Box 197 Glen Osmond, SA, 5064, Australia

[2]ARC Centre of Excellence in Synthetic Biology and Department of Molecular Sciences

[3]New South Wales Department of Primary Industries, Elizabeth Macarthur Agricultural Institute, Woodbridge Road, Menangle, NSW 2568

[4]The Chancellery, Macquarie University, Sydney, NSW 2109, Australia.

[5] Current address, The Chancellery, Macquarie University, Sydney, NSW 2109, Australia.

[6]School of Wine, Food and Agriculture, The University of Adelaide, South Australia, 5005. Australia

*Correspondence to: anthony.borneman@awri.com.au

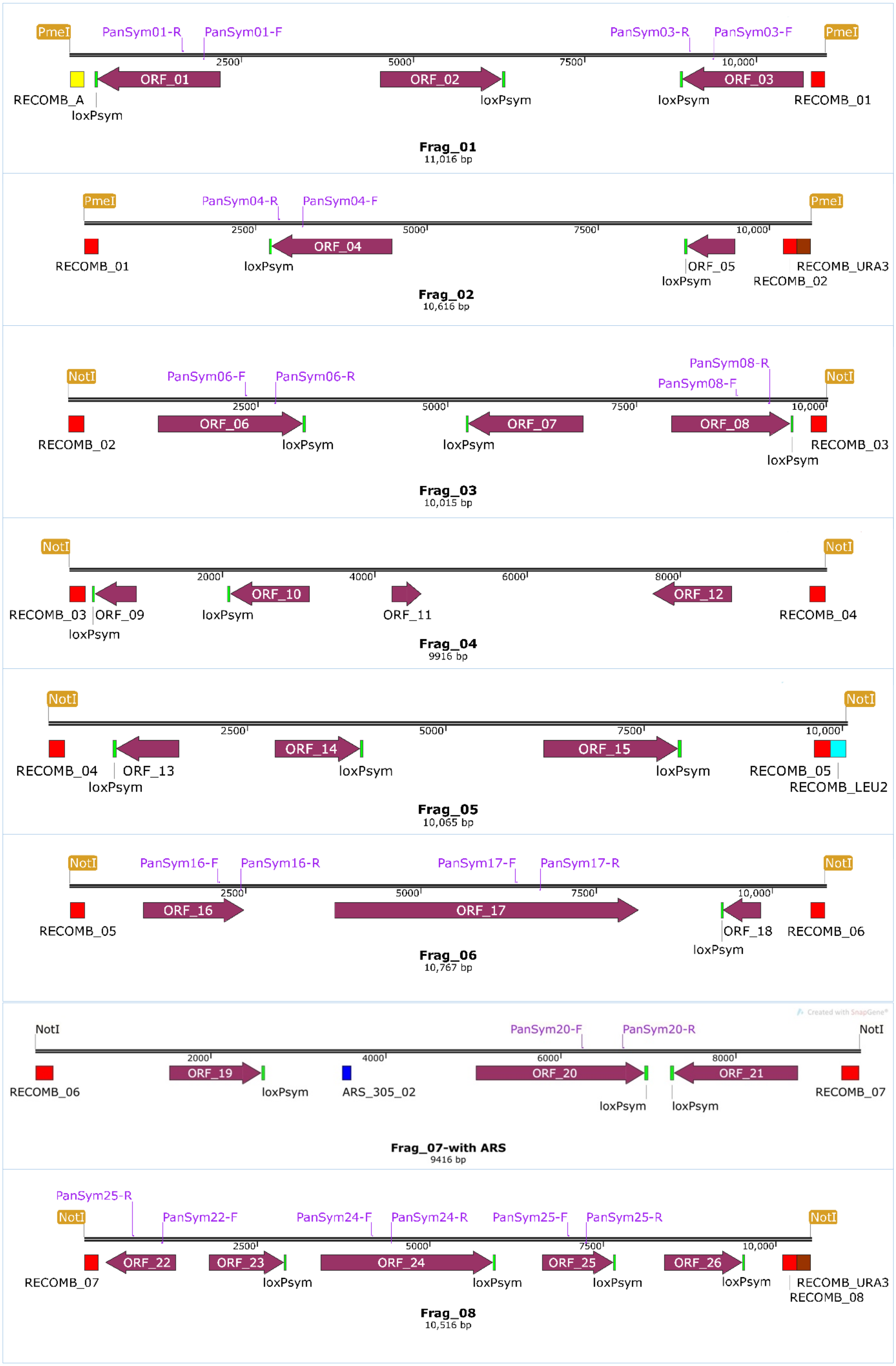
PmeI
PanSym01-R
PanSym01-F
PanSym03-R
PanSym03-F
PmeI
2500
5000
7500
10,000
ORF_01
ORF_02
ORF_03
RECOMB_A
loxPsym
loxPsym
loxPsym
RECOMB_01
Frag_01
11,016 bp
PmeI
PanSym04-R
PanSym04-F
PmeI
2500
5000
7500
10,000
ORF_04
RECOMB_01
loxPsym
ORF_05
loxPsym
RECOMB_URA3
RECOMB_02
Frag_02
10,616 bp
NotI
PanSym06-F
PanSym06-R
PanSym08-R
PanSym08-F
NotI
2500
5000
7500
10,000
ORF_06
ORF_07
ORF_08
RECOMB_02
loxPsym
loxPsym
RECOMB_03
loxPsym
Frag_03
10,015 bp
NotI
NotI
2000
4000
6000
8000
ORF_10
ORF_12
RECOMB_03
ORF_09
loxPsym
loxPsym
ORF_11
RECOMB_04
Frag_04
9916 bp
NotI
NotI
2500
5000
7500
10,000
ORF_14
ORF_15
RECOMB_04
ORF_13
loxPsym
loxPsym
loxPsym
RECOMB_05
RECOMB_LEU2
Frag_05
10,065 bp
NotI
PanSym16-F
PanSym16-R
PanSym17-F
PanSym17-R
NotI
2500
5000
7500
10,000
ORF_16
ORF_17
RECOMB_05
ORF_18
RECOMB_06
loxPsym
Frag_06
10,767 bp
Created with SnapGene®
NotI
PanSym20-F
PanSym20-R
NotI
2000
4000
6000
8000
ORF_19
ORF_20
ORF_21
RECOMB_06
loxPsym
ARS_305_02
loxPsym
loxPsym
RECOMB_07
Frag_07-with ARS
9416 bp
PanSym25-R
NotI
PanSym22-F
PanSym24-F
PanSym24-R
PanSym25-F
PanSym25-R
NotI
2500
5000
7500
10,000
ORF_22
ORF_23
ORF_24
ORF_25
ORF_26
RECOMB_07
loxPsym
loxPsym
loxPsym
loxPsym
RECOMB_URA3
RECOMB_08
Frag_08
10,516 bp

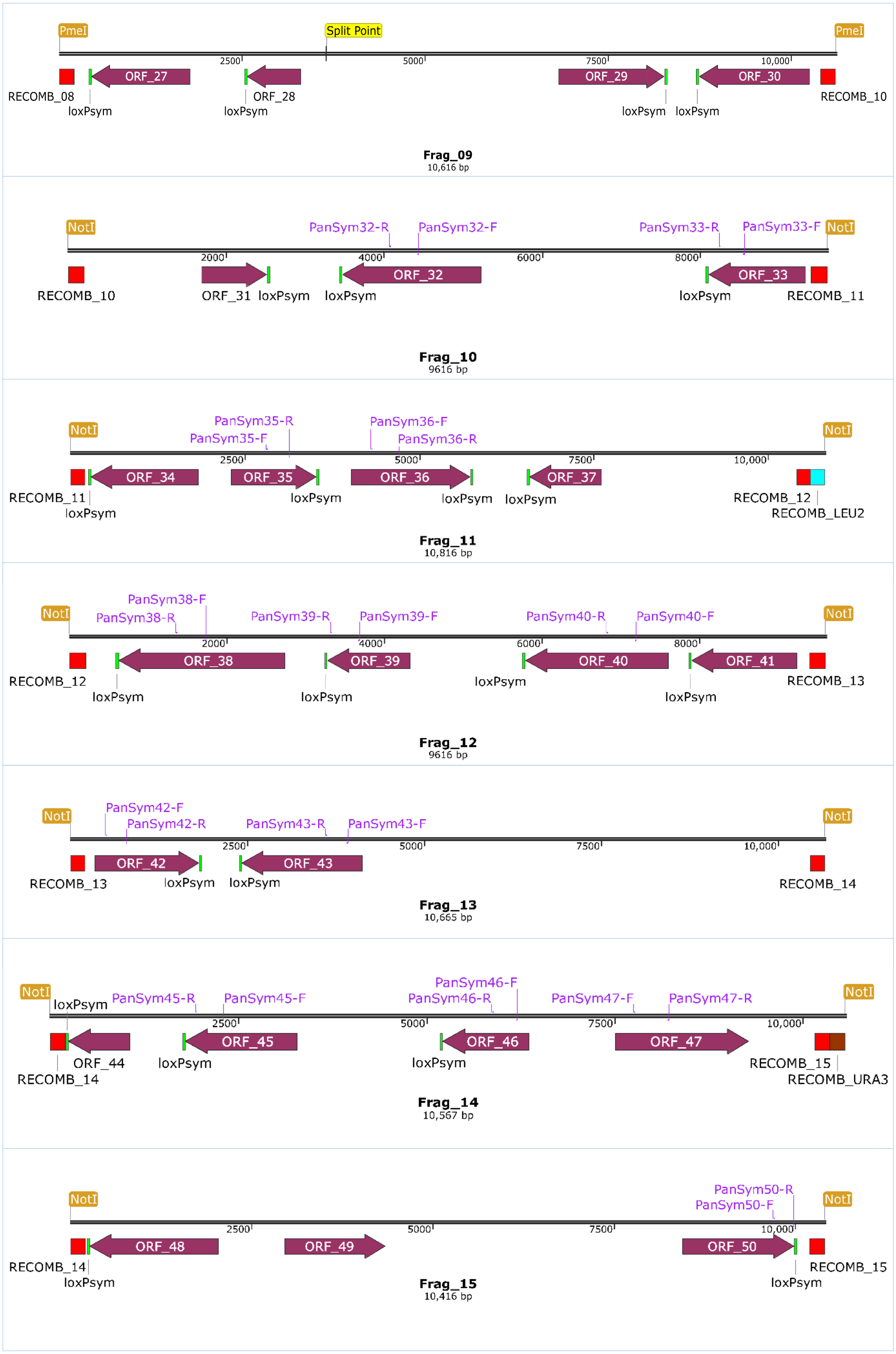
PmeI
Split Point
PmeI
2500
5000
7500
10,000
ORF_27
ORF_29
ORF_30
RECOMB_08
loxPsym
ORF_28
loxPsym
loxPsym
loxPsym
RECOMB_10
Frag_09
10,616 bp
NotI
PanSym32-R
PanSym32-F
PanSym33-R
PanSym33-F
NotI
2000
4000
6000
8000
ORF_32
ORF_33
RECOMB_10
ORF_31
loxPsym
loxPsym
loxPsym
RECOMB_11
Frag_10
9616 bp
NotI
PanSym35-R
PanSym35-F
PanSym36-F
PanSym36-R
NotI
2500
5000
7500
10,000
ORF_34
ORF_35
ORF_36
ORF_37
RECOMB_11
loxPsym
loxPsym
loxPsym
loxPsym
RECOMB_12
RECOMB_LEU2
Frag_11
10,816 bp
PanSym38-F
NotI
PanSym38-R
PanSym39-R
PanSym39-F
PanSym40-R
PanSym40-F
NotI
2000
4000
6000
8000
ORF_38
ORF_39
ORF_40
ORF_41
RECOMB_12
loxPsym
loxPsym
loxPsym
loxPsym
RECOMB_13
Frag_12
9616 bp
PanSym42-F
NotI
PanSym42-R
PanSym43-R
PanSym43-F
NotI
2500
5000
7500
10,000
ORF_42
ORF_43
RECOMB_13
loxPsym
loxPsym
RECOMB_14
Frag_13
10,665 bp
PanSym46-F
NotI
loxPsym
PanSym45-R
PanSym45-F
PanSym46-R
PanSym47-F
PanSym47-R
NotI
2500
5000
7500
10,000
ORF_45
ORF_46
ORF_47
ORF_44
loxPsym
loxPsym
RECOMB_14
RECOMB_15
RECOMB_URA3
Frag_14
10,567 bp
PanSym50-R
NotI
PanSym50-F
NotI
2500
5000
7500
10,000
ORF_48
ORF_49
ORF_50
RECOMB_14
loxPsym
RECOMB_15
loxPsym
Frag_15
10,416 bp

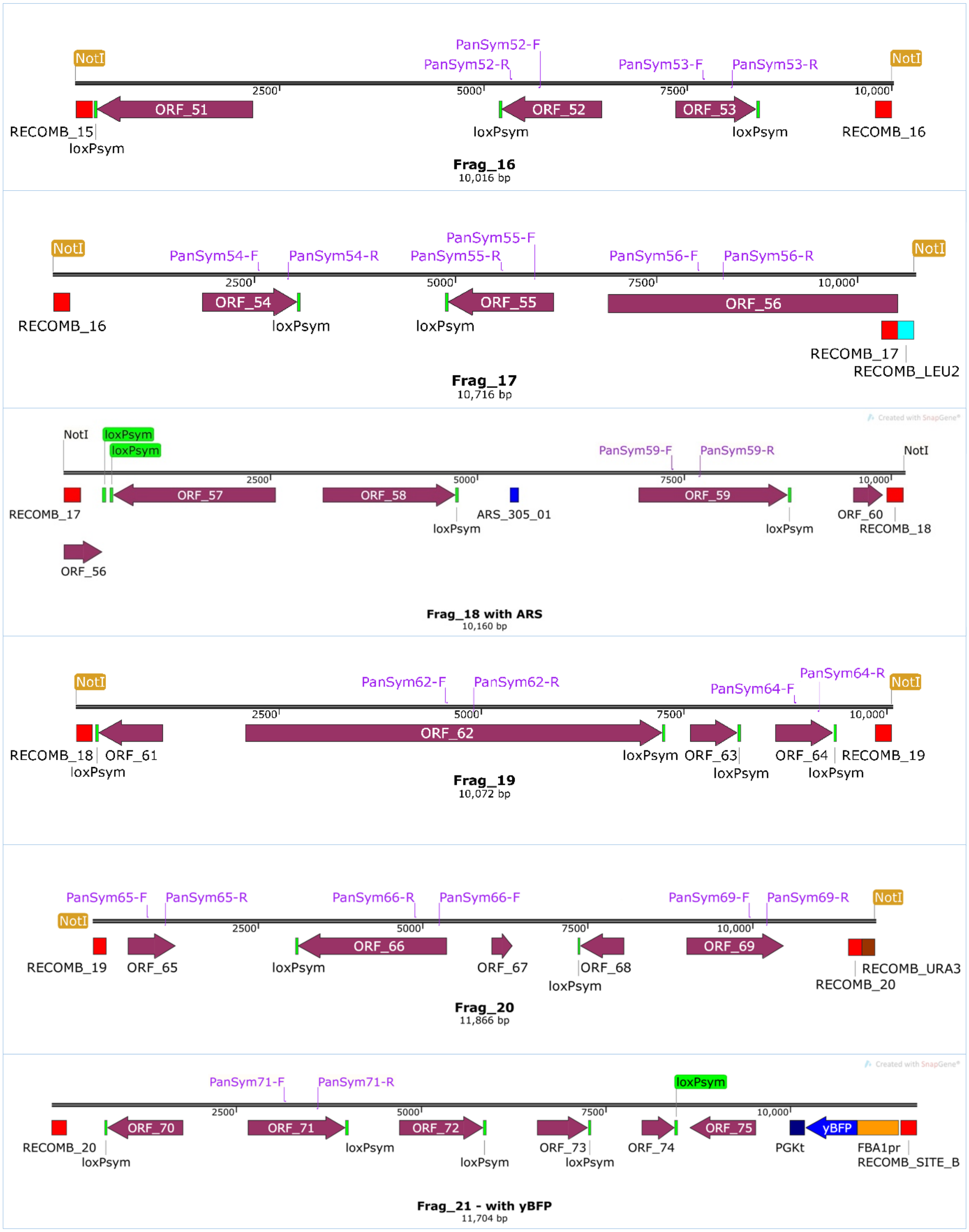

Supplementary Fig. 1. Maps of 22 synthetic DNA fragments that were used to construct the PGNC. Fragment 9 was divided into two overlapping fragments as indicated by "split point" to aid in assembly. ARS_305_01 and ARS_305_02 mark the positions of ARS305 insertions for the stability experiments.

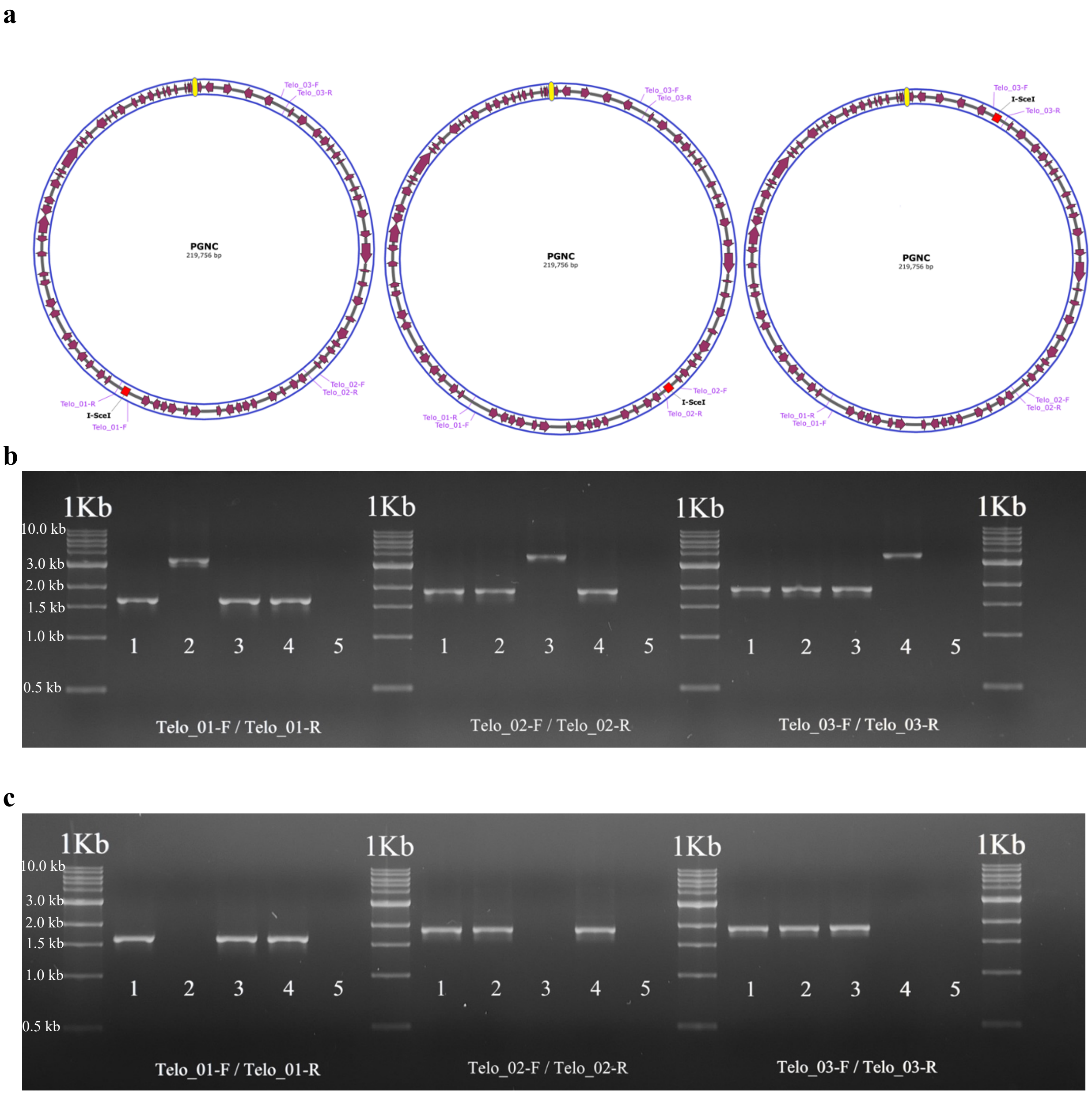

Supplementary Fig. 2. Linearization of the pan-genome neo-chromosome. a Schematic representation of the various PGNC configurations with the respective positions of the telomerator insertion shown (red) relative to the PGNC centromere (yellow). **b** Confirmation of integration of the telomerator element into the PGNC^{circ} (1) at three different sites (PGNC^{lin1}, 2), (PGNC^{lin2}, 3) and (PGNC^{lin3}, 4). The expected amplicon size for the successful integration of the telomerator using Telo_01-F/R, Telo_02-F/R and Telo_03-F/R primer set are 3196 bp, 3418bp and 3474 bp respectively. Expected control amplicon sizes are 1631 bp, 1853 bp and 1909 bp respectively. A DNA negative control is presented in line 5. **c** Confirmation of successful linearization of the telomerator variants. Strains and expected sizes are as for **b**. Loss of the telomerator band is due to the linearization of the element. PCR reactions in **b** and **c** were confirmed using at least two independent experiments.

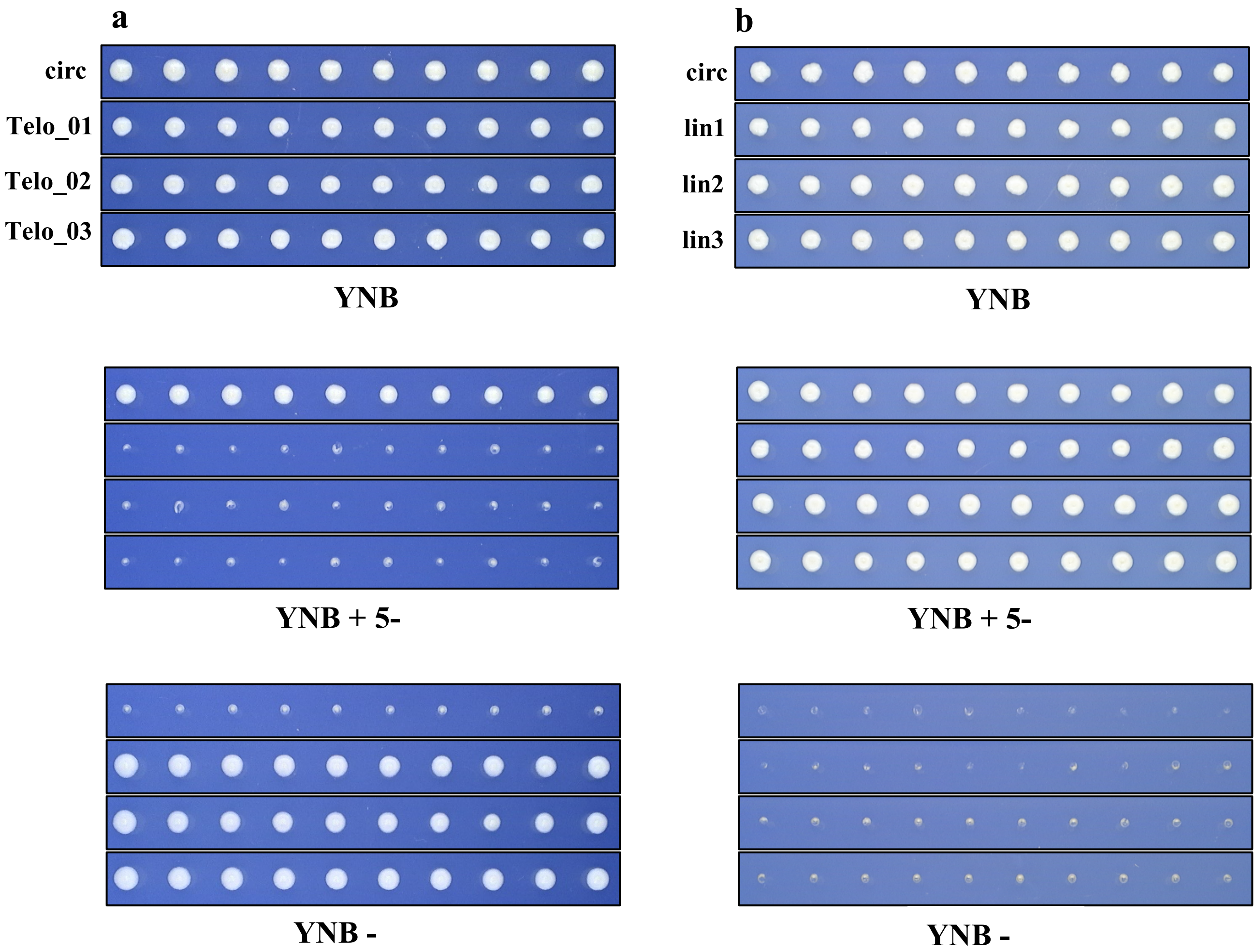

Supplementary Fig. 3. Growth of the PGNC variants with integrated telomerators, and after the linearization. 10 colonies of PGNC^{circ} and variants carrying the telomerator in three different integration sites (PGNC^{Telo_01}, PGNC^{Telo_02} and PGNC^{Telo_03}) (a), as well as 10 colonies of PGNC^{circ} and three different linearized variants (PGNC^{lin1}, PGNC^{lin2} and PGNC^{lin3}) (b), were pinned onto solid YNB, YNB + 1g/L of 5-FOA and YNB lacking Uracil media, all containing 100 µg/mL of ClonNAT. Photographs taken after 72 h incubation at 30°C.

AWRI4300(PGNC^{circ}) - YPD

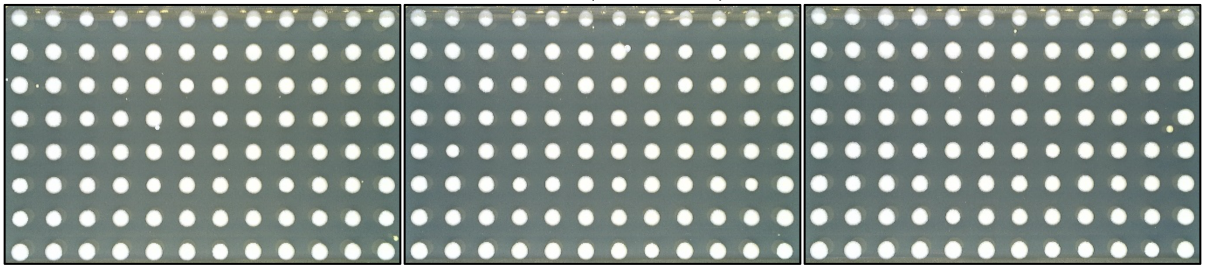

AWRI4300(PGNC^{circ}) – YPD+ClonNAT

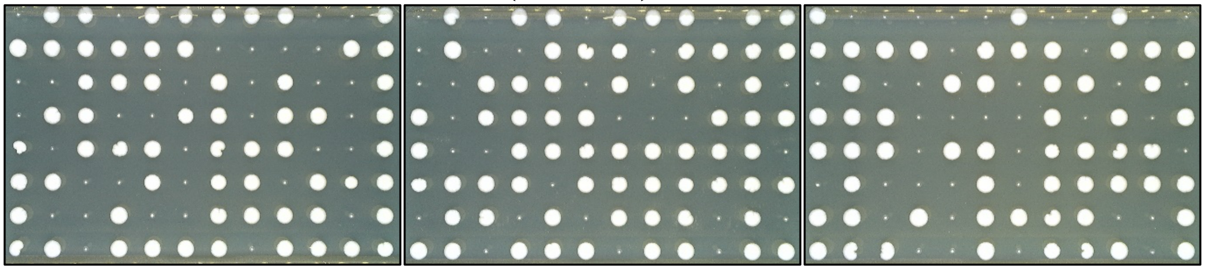

AWRI4301(PGNC^{lin1}) - YPD

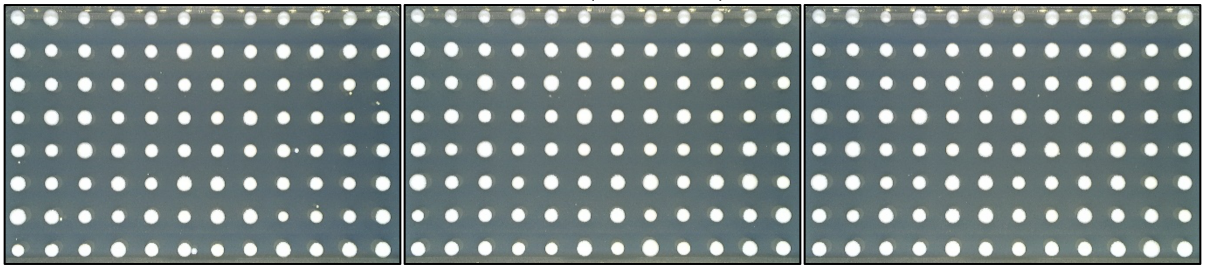

AWRI4301(PGNC^{lin1}) – YPD+ClonNAT

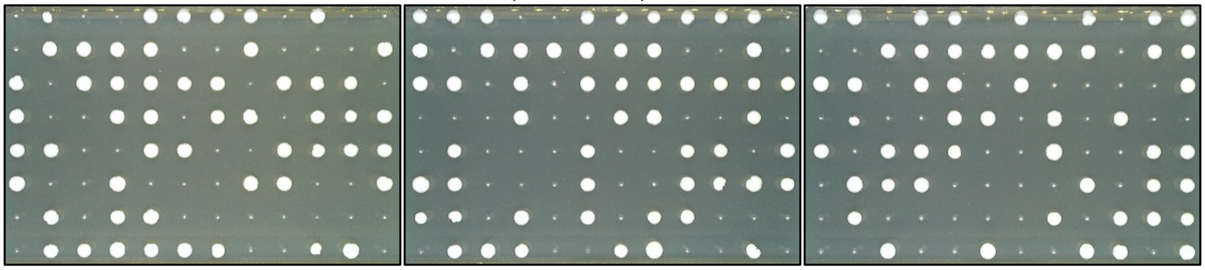

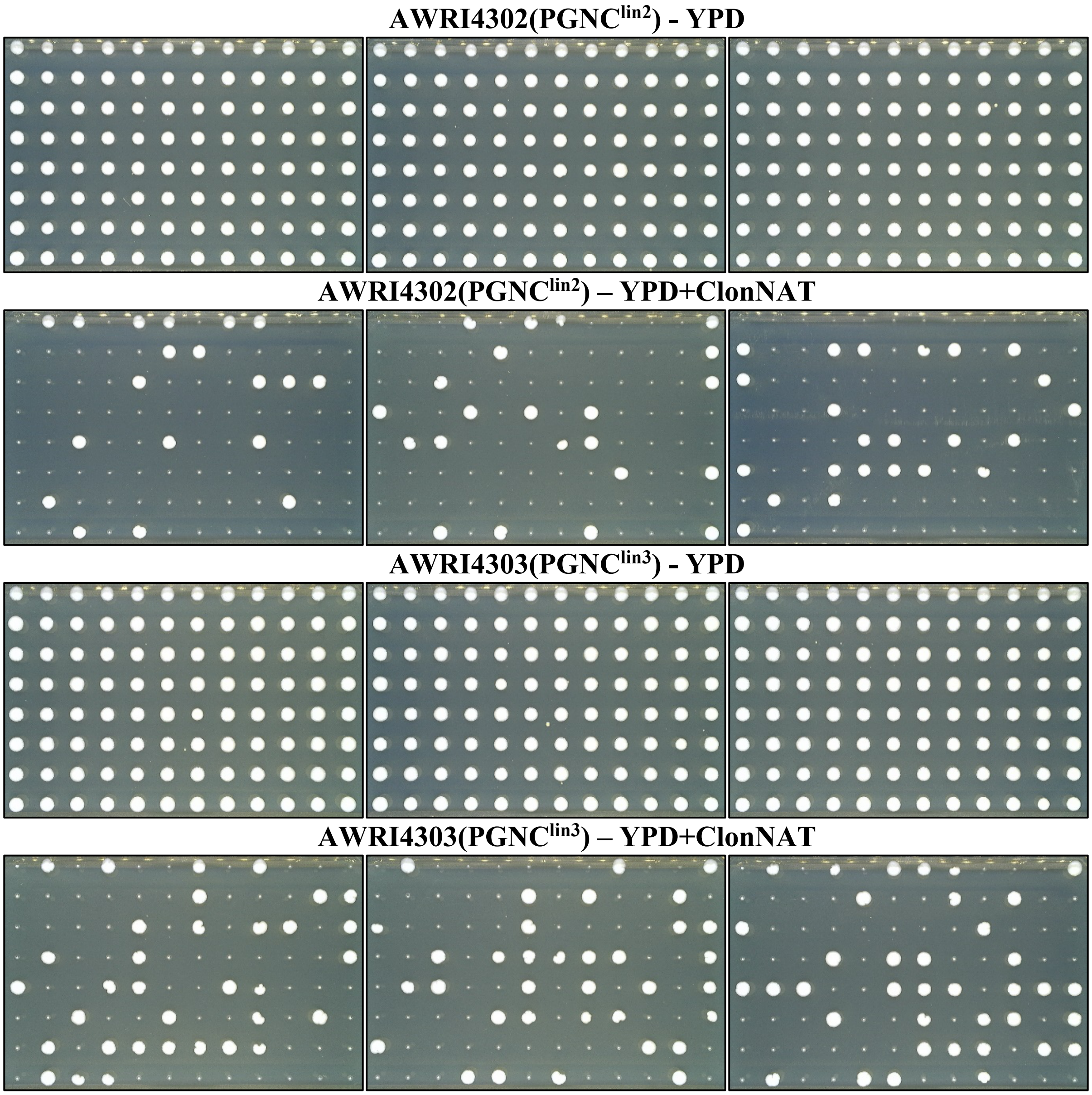

Supplementary Fig. 4. PGNC stability in the absence of 25 generations of selection. Colonies from triplicate populations of AWRI4300, 4301, 4302 and 4303 after 25 generations growth in non-selective medium (YPD without ClonNAT). Colonies were pinned onto YPD and YPD + 100 µg/mL of ClonNAT. Photographs taken after 72 h incubation at 30°C.

AWRI4300(PGNC^{circ}) - YPD

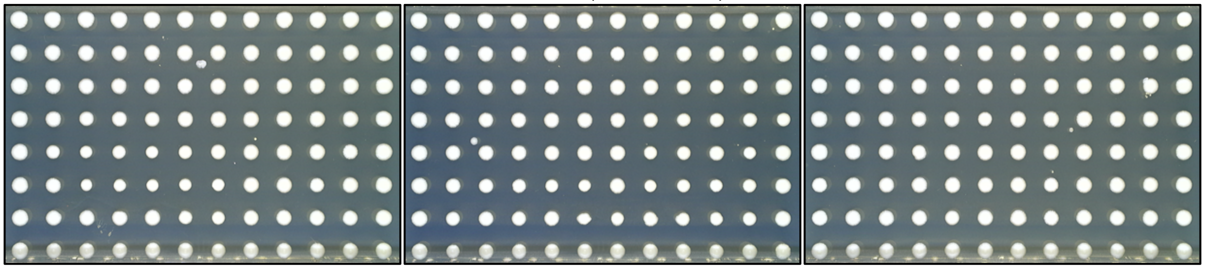

AWRI4300(PGNC^{circ}) – YPD+ClonNAT

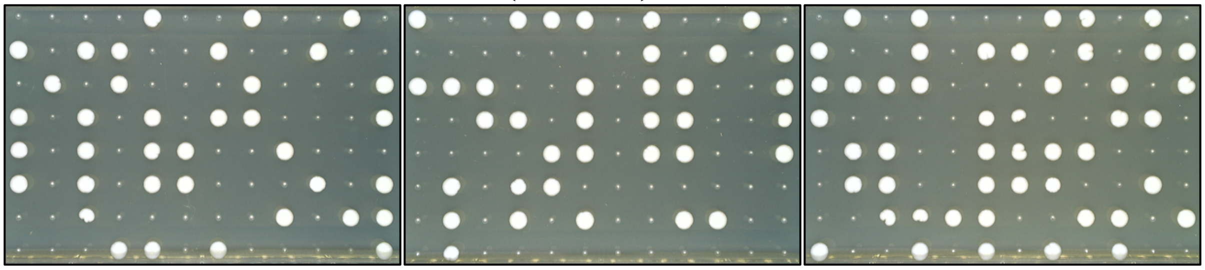

AWRI4301(PGNC^{lin1}) - YPD

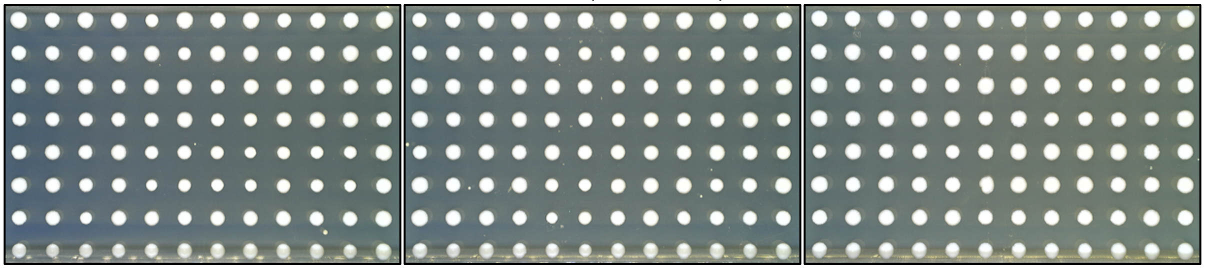

AWRI4301(PGNC ^{lin1}) – YPD+ClonNAT

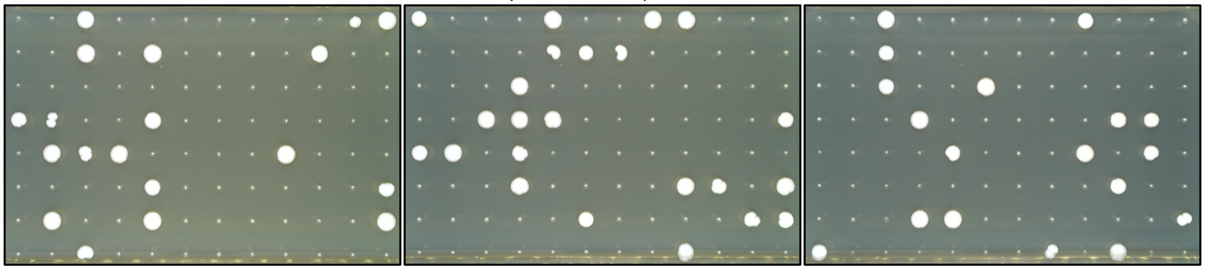

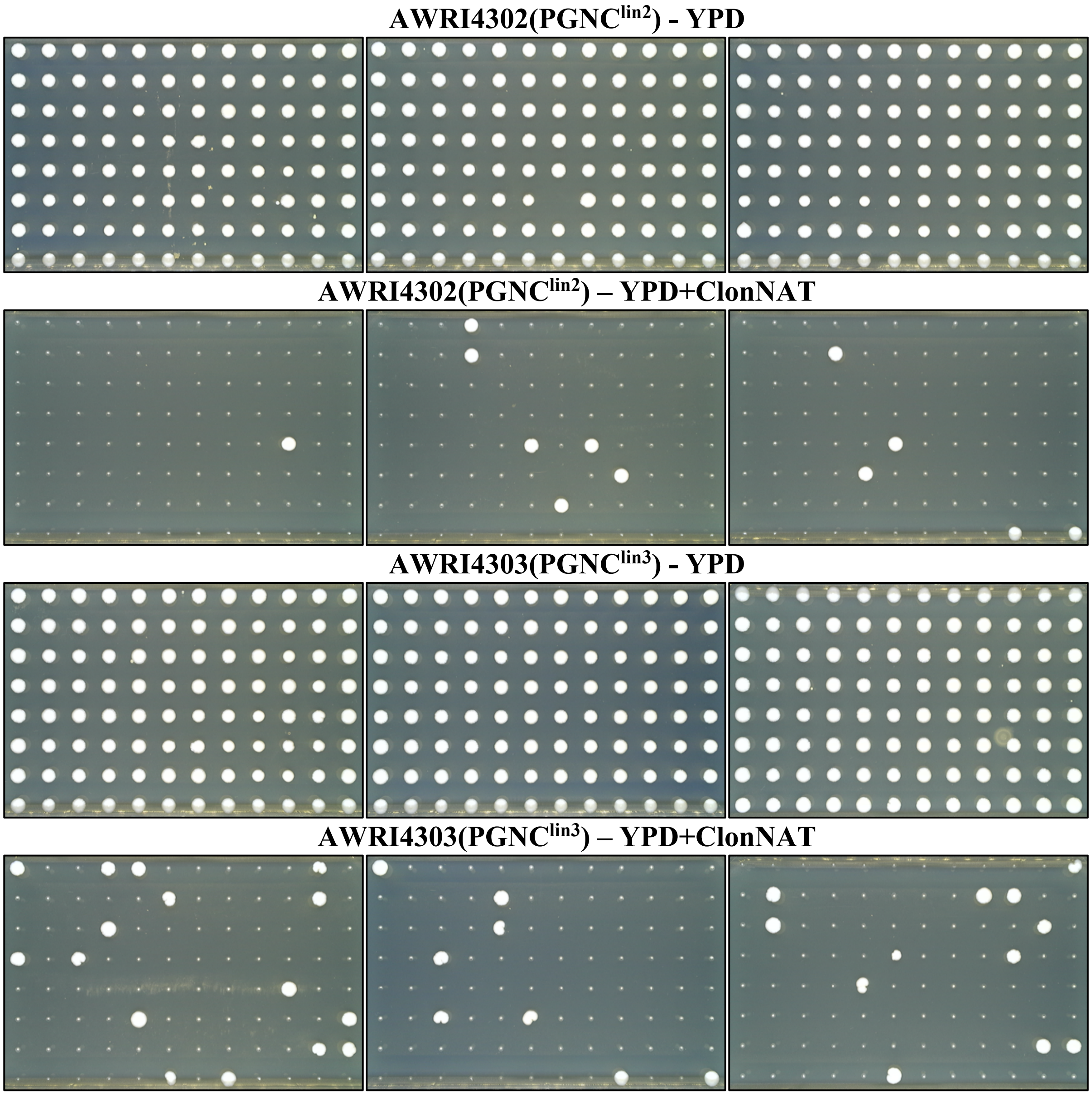

Supplementary Fig. 5. PGNC stability in the absence of 50 generations of selection. Colonies from triplicate populations of AWRI4300, 4301, 4302 and 4303 after 50 generations growth in non-selective medium (YPD without ClonNAT). Colonies were pinned onto YPD and YPD + 100 µg/mL of ClonNAT. Photographs taken after 72 h incubation at 30°C.

Supplementary Table 1. PGNC watermark locations

ORF	5' watermark	PGNC position	3' water mark	PGNC position
PanSym0001	PanSym0001-R	1429..1449	PanSym0001-F	complement(1730..1751)
PanSym0003	PanSym0003-R	8826..8846	PanSym0003-F	complement(9156..9178)
PanSym0004	PanSym0004-R	13430..13450	PanSym0004-F	complement(13760..13782)
PanSym0006	PanSym0006-F	23133..23153	PanSym0006-R	complement(23511..23531)
PanSym0008	PanSym0008-F	29610..29631	PanSym0008-R	complement(30027..30046)
PanSym0016	PanSym0016-F	52055..52077	PanSym0016-R	complement(52358..52378)
PanSym0017	PanSym0017-F	56296..56316	PanSym0017-R	complement(56629..56649)
PanSym0020	PanSym0020-F	66633..66653	PanSym0020-R	Complement(67098..67119)
PanSym0022	PanSym0022-R	70294..70312	PanSym0022-F	complement(70706..70728)
PanSym0024	PanSym0024-F	73752..73773	PanSym0024-R	complement(74020..74040)
PanSym0025	PanSym0025-F	76587..76608	PanSym0025-R	complement(76833..76851)
PanSym0032	PanSym0032-R	94167..94188	PanSym0032-F	complement(94512..94534)
PanSym0033	PanSym0033-R	98339..98358	PanSym0033-F	complement(98637..98657)
PanSym0035	PanSym0035-F	102301..102321	PanSym0035-R	complement(102617..102638)
PanSym0036	PanSym0036-F	103800..103819	PanSym0036-R	complement(104188..104208)
PanSym0038	PanSym0038-R	111246..111268	PanSym0038-F	complement(111606..111625)
PanSym0039	PanSym0039-R	113210..113230	PanSym0039-F	complement(113558..113579)
PanSym0040	PanSym0040-R	116718..116738	PanSym0040-F	complement(117074..117094)
PanSym0042	PanSym0042-F	119793..119810	PanSym0042-R	complement(120074..120096)
PanSym0043	PanSym0043-R	122903..122923	PanSym0043-F	complement(123196..123216)
PanSym0045	PanSym0045-R	131680..131700	PanSym0045-F	complement(132034..132054)
PanSym0046	PanSym0046-R	135614..135633	PanSym0046-F	complement(135931..135952)
PanSym0047	PanSym0047-F	137497..137517	PanSym0047-R	complement(137943..137966)
PanSym0050	PanSym0050-F	149595..149615	PanSym0050-R	complement(149856..149879)
PanSym0052	PanSym0052-R	155420..155441	PanSym0052-F	complement(155768..155788)
PanSym0053	PanSym0053-F	157787..157807	PanSym0053-R	complement(158125..158146)
PanSym0054	PanSym0054-F	162446..162466	PanSym0054-R	complement(162799..162820)
PanSym0055	PanSym0055-R	165465..165487	PanSym0055-F	complement(165867..165887)
PanSym0056	PanSym0056-F	167910..167929	PanSym0056-R	complement(168216..168233)
PanSym0059	PanSym0059-F	177441..177461	PanSym0059-R	complement(177763..177783)
PanSym0062	PanSym0062-F	184604..184624	PanSym0062-R	complement(184929..184949)
PanSym0064	PanSym0064-F	188909..188929	PanSym0064-R	complement(189187..189208)
PanSym0065	PanSym0065-F	190720..190740	PanSym0065-R	complement(190975..190997)
PanSym0066	PanSym0066-R	194781..194799	PanSym0066-F	complement(195130..195148)
PanSym0069	PanSym0069-F	199849..199868	PanSym0069-R	complement(200099..200121)
PanSym0071	PanSym0071-F	204500..204520	PanSym0071-R	complement(204936..204957)

Supplementary Table 2. Structural variation observed in SCRaMbLEd isolates

SCRaMbLEant	PGNC position	Size (bp)	SV type
1	n.d		
2	n.d		
3	n.d		
4	n.d		
5	67355..67650	295	deletion
	75550..117760	42209	deletion
6	n.d		
7	n.d		
8	67389..67650	261	inversion
	174968..187276	12308	deletion
9	n.d		
10	n.d		
11	113171..121681	8510	deletion
12	207200..208621	1421	deletion

n.d: none detected

Supplementary Table 3. Primers used to amplify inter-chunk junctions

Locus	Primer	Primer sequence	PCR product size (bp)
Pair_01	V/FR1-F	AAAGGATCTAGGTGAAGATCCT	693
	V/FR1-R	AAATTCGCATTAAGTTCGTCC	
Pair_02	FR1/FR2-F	TTAAAAAGAACACGGAATATGCC	1304
	FR1/FR2-R	TTCGACATCGTAAATATATGGGC	
Pair_03	FR2/FR3-F	TTCAAATGAGAGGTGATTTGAGC	883
	FR2/FR3-R	GACAGGCAAGATATTGAGCA	
Pair_04	FR3/FR4-F	TATGGCTATTTCAACTGCCTTC	1085
	FR3/FR4-R	TTCAGGATTATCGGCATATGC	
Pair_05	FR4/FR5-F	CAGAAGTATATGTGGACTTTCCT	863
	FR4/FR5-R	GAGTAAAGTGCAGTATTGGATGAA	
Pair_06	FR5/FR6-F	TACTGTGGTGATGATAGAGATTCG	1337
	FR5/FR6-R	GTACCTGAACTATGTCTACATCC	
Pair_07	FR6/FR7-F	GAAGTGAGAGTTGATTGCAGTAA	1414
	FR6/FR7-R	CAGAAACAAAGCCCTATTATCC	
Pair_08	FR7/FR8-F	CACATTAATTTCGTACAGACACG	823
	FR7/FR8-R	CCAAGGAATCAAGGCGATATTAT	
Pair_09	FR8/FR9-F	CGAAACCACTTTGAGGAATAGAA	1114
	FR8/FR9-R	TCTGATTAGTGGCAATTCTCATAGT	
Pair_10	FR9/FR10-F	CAATGACAGCTCTTTTATCATCG	1262
	FR9/FR10-R	CTGATGTACCCTTGTGAAATCTAA	
Pair_11	FR10/FR11-F	TTAGTGATCAAAGGCTCTATAACG	1451
	FR10/FR11-R	TCGTCCGAGTATTGCAAATAATC	
Pair_12	FR11/FR12-F	CTGTCACATCTACTAACTGTACTAC	1219
	FR11/FR12-R	AGTCATATTGATTGGAGTAGCTTC	
Pair_13	FR12/FR13-F	CGATAGTAAGAAGCAACTCTTGA	911
	FR12/FR13-R	TATGATCTGGTAGGCATCTACTTC	
Pair_14	FR13/FR14-F	CTCTAGGTCTTTCAGCAATAATGT	1141
	FR13/FR14-R	TTAATGAAACTTAGGTCTGTGGC	
Pair_15	FR14/FR15-F	TATCTGCAATACGATGGAGAATTG	853
	FR14/FR15-R	TCTAATTTCGACATCTTCGTACTTC	
Pair_16	FR15/FR16-F	TAAGGGTGCAGGTAATTGTATGA	1031
	FR15/FR16-R	CAGTAGTTAGGGATTCGAAAAATG	
Pair_17	FR16/FR17-F	TAATACAACACATCACCCAATAGC	1102
	FR16/FR17-R	ACAACTAGCAAGGTAAGAGTATAC	
Pair_18	FR17/FR18-F	GGCTCTATCCTAAGTCCTATCAA	1241
	FR17/FR18-R	CACCATTCGACCTTCGAAATAAA	
Pair_19	FR18/FR19-F	CATGGCTACAGAAAATAATTCGAG	882
	FR18/FR19-R	GAAATTGAATACTGTCCATCCTG	
Pair_20	FR19/FR20-F	CTAGCAGAGGGATTTACTAATTCT	1022
	FR19/FR20-R	CGATTCCAAATCAGATACATCGA	
Pair_21	FR20/FR21-F	CAGAACATATTGAAGAGTCCAAAC	1320
	FR20/FR21-R	TACATTTTGGGACACATTGATTGG	
Pair_22	FR21/V-F	TATACTAGAAGTTCTCCTCCAGGA	1916
	FR21/V-R	CCGACAGGACTATAAAGATACCA	

Supplementary Table 4. Primers used for the replacement of *LEU2* with *BFP2*.

PCR	Primer	Primer sequence
Pair 1	Del-LEU2-C7-NEO8-F	ATTCTCAAAATGGAAACTACACTTCTACATACTAATATTACGATTATTCCgttttttctccttgacgttaaagtat
	Del-LEU2-C7-NEO8-R	CTATCGTCTTGAGTCCAACCCGGTAAGACACGACTTATCGCCACTGGCAGctcgaggtcgacggtatcgat
Pair 2	C7-repl-BFP-NEO8-F	ATTCTCAAAATGGAAACTACACTTCTACATACTAATATTACGATTATTCCtgacagcaggattatcgtaatacg
	C7-repl-BFP-NEO8-R	CTATCGTCTTGAGTCCAACCCGGTAAGACACGACTTATCGCCACTGGCAGacagatctctagacctagatagctt

Sequences complementary to the location on the PGNC are depicted in red. Primers Pair 1 was used to amplify the CORE7 cassette from pCORE7 vector. Primers Pair 2 was used to amplify BFP2 cassette from pCV2-BFP2 vector.

Supplementary Table 5. Primers used for the removal of the BY4742 auxotrophic markers

Locus	Primers	Primer sequence
HIS3	HIS3-F	AGCGATTGGCATTATCACATAATG
	HIS3-R	CATTACCTTGTCATCTTCAGTATCA
LEU2	LEU2-F	GATGACAAAACCTCTTCCGATAAA
	LEU2-R	CCATTCTTCCATCAGATTTGGTATTG
LYS2	LYS2-F	CTTTGAAGTATTGGCTACTTCAAG
	LYS2-R	GCTTCGCAAGTATTCATTTTAGAC
URA3	URA3-F	CAGACGCTCATCAATAAAATCGAA
	URA3-R	AGCCCATATCCAACTTCCAATTTA

Supplementary Table 6. Telomerator insertion primers.

PCR	Primer	Primer sequence
Ins_01	Tel_ampl_01-F	TCGCGACGAAGGTGGATGGGAAACCCAGAATAATATCAGCATAGCATAATttcaattcatcatttttttttt
	Tel_ampl_01-R	TCGGTCTCAATGATCGGGGTGTAATACGTTGATCAAACCTAGTGGGATACcctgatgcggtattttctcc
Ins_02	Tel_ampl_02-F	TGGCTACTATCGAAAGATTCTAGTAATGTAAATTGCTTGGTCACGAGAAAttcaattcatcatttttttttt
	Tel_ampl_02-R	TATGGAATACTTTTCTTACCTGTCTTCAGAAAAAATCTAAGGTGTTTTGAcctgatgcggtattttctcc
Ins_03	Tel_ampl_03-F	TGATCGATTTATGAAGCATTACGAGCAAGGTTCACTCAGTTTAATATTTGttcaattcatcatttttttttt
	Tel_ampl_03-R	ATCAAAAGTTGAGTATTGAAAAATATGCTTTAGAAAATCTGTAACTTATAcctgatgcggtattttctcc

Sequences complementary to the location on the PGNC are depicted in red.

Supplementary Table 7. Telomerator confirmation primers

Locus	Primer	Primer sequence
**Telomerator_01**	Telo_01-F	AATAGACCTAGTCATCCAGCAAAA
	Telo_01-R	AGAAGAAATCTCTCAGATCCCAG
**Telomerator_02**	Telo_02-F	TAAACTATCCATGTAACAACCGGT
	Telo_02-R	AGATGAATGCTAAGCCAAAGGTAA
**Telomerator_03**	Telo_03-F	TGACATGTAATACAGGCAGTGGA
	Telo_03-R	CCCCAGGATGTCTATCAATTACT
