# Supplementary material for: Construction of a synthetic Saccharomyces cerevisiae pan-genome neo-chromosome
Source: Nat Commun. 2022 Jun 24;13:3628. doi: 10.1038/s41467-022-31305-4 (PMC9232646; doi:10.1038/s41467-022-31305-4)
Supplement: Supplementary file 2 — Reporting Summary [file 41467_2022_31305_MOESM2_ESM.pdf]

nature portfolio

Corresponding author(s): Anthony Borneman

Last updated by author(s): Jun 8, 2022

Reporting Summary

Nature Portfolio wishes to improve the reproducibility of the work that we publish. This form provides structure for consistency and transparency in reporting. For further information on Nature Portfolio policies, see our Editorial Policies and the Editorial Policy Checklist.

Statistics

For all statistical analyses, confirm that the following items are present in the figure legend, table legend, main text, or Methods section.

n/a	Confirmed	
☐	☒	The exact sample size (*n*) for each experimental group/condition, given as a discrete number and unit of measurement
☐	☒	A statement on whether measurements were taken from distinct samples or whether the same sample was measured repeatedly
☒	☐	The statistical test(s) used AND whether they are one- or two-sided<br>*Only common tests should be described solely by name; describe more complex techniques in the Methods section.*
☒	☐	A description of all covariates tested
☒	☐	A description of any assumptions or corrections, such as tests of normality and adjustment for multiple comparisons
☐	☒	A full description of the statistical parameters including central tendency (e.g. means) or other basic estimates (e.g. regression coefficient) AND variation (e.g. standard deviation) or associated estimates of uncertainty (e.g. confidence intervals)
☒	☐	For null hypothesis testing, the test statistic (e.g. *F*, *t*, *r*) with confidence intervals, effect sizes, degrees of freedom and *P* value noted<br>*Give P values as exact values whenever suitable.*
☒	☐	For Bayesian analysis, information on the choice of priors and Markov chain Monte Carlo settings
☒	☐	For hierarchical and complex designs, identification of the appropriate level for tests and full reporting of outcomes
☒	☐	Estimates of effect sizes (e.g. Cohen's *d*, Pearson's *r*), indicating how they were calculated

Our web collection on statistics for biologists contains articles on many of the points above.

Software and code

Policy information about availability of computer code

Data collection	Sequencing data were obtained using MinKnow(v19.10.1)
Data analysis	Raw BioLog data was analyzed using the R package opm (v1.3.77)<br>Assemblies and analysis were performed with Guppy(v.3.2.1), Canu(v.1.7.1), Nanopolish(v.0.11.2), Minimap2(v.2.17) and CoverM(v.0.4.0).<br>Functional annotation was performed using the Interproscan 5 pipeline v. 5.52-86.0.<br>Annotation of Carbohydrate-active enzyme (CAZYme) classes was performed through an HMMer search v.3.3.2 of the dbCAN HMMdb v.9.0 database.<br>KEGG orthology assignments were obtained using BlastKOALA v.2.2 and prediction of signal peptides was performed using SignalP v.4.1

For manuscripts utilizing custom algorithms or software that are central to the research but not yet described in published literature, software must be made available to editors and reviewers. We strongly encourage code deposition in a community repository (e.g. GitHub). See the Nature Portfolio guidelines for submitting code & software for further information.

Data

Policy information about availability of data

All manuscripts must include a data availability statement. This statement should provide the following information, where applicable:

- Accession codes, unique identifiers, or web links for publicly available datasets
- A description of any restrictions on data availability
- For clinical datasets or third party data, please ensure that the statement adheres to our policy

The strains and plasmids generated in this study have been deposited and are available from the Australian Wine Research Institute Culture Collection [https://www.awri.com.au/research_and_development/wine-microorganism/]. The raw DNA sequence data generated in this study have been deposited under BioProject

accession code PRJNA615683 [https://www.ncbi.nlm.nih.gov/bioproject/PRJNA615683]. All other data generated in this study are provided in the Supplementary Information/Source Data files.

Field-specific reporting

Please select the one below that is the best fit for your research. If you are not sure, read the appropriate sections before making your selection.

☒ Life sciences ☐ Behavioural & social sciences ☐ Ecological, evolutionary & environmental sciences

For a reference copy of the document with all sections, see nature.com/documents/nr-reporting-summary-flat.pdf

Life sciences study design

All studies must disclose on these points even when the disclosure is negative.

Sample size	Biological triplicates were chosen for sample sizes for all growth assays, as is standard for controlled microbial growth studies. However effect size and variability were taken into account in deciding if additional samples were required beyond the triplicates used.
Data exclusions	Data from all replicates were included, no data were excluded
Replication	Growth determinations were all conducted using biological triplicates at a minimum with all data reported. For gel photos, data were repeated at least twice, with similar results obtained for each iteration.
Randomization	Replicates were randomized across microplate growth assays to avoid position effects. No randomization of isolate could be performed as the only covariate was the genotype of the neo-chromosome that was being assessed.
Blinding	No group allocation was conducted as it was not possible to externally influence the growth of one microbial culture relative to others.

Reporting for specific materials, systems and methods

We require information from authors about some types of materials, experimental systems and methods used in many studies. Here, indicate whether each material, system or method listed is relevant to your study. If you are not sure if a list item applies to your research, read the appropriate section before selecting a response.

Materials & experimental systems

n/a	Involved in the study
☒	☐ Antibodies
☒	☐ Eukaryotic cell lines
☒	☐ Palaeontology and archaeology
☒	☐ Animals and other organisms
☒	☐ Human research participants
☒	☐ Clinical data
☒	☐ Dual use research of concern

Methods

n/a	Involved in the study
☒	☐ ChIP-seq
☒	☐ Flow cytometry
☒	☐ MRI-based neuroimaging
