# Supplementary material for: Construction of a synthetic Saccharomyces cerevisiae pan-genome neo-chromosome
Source: Nat Commun. 2022 Jun 24;13:3628. doi: 10.1038/s41467-022-31305-4 (PMC9232646; doi:10.1038/s41467-022-31305-4)
Supplement: Supplementary file 3 — Description of Additional Supplementary Files [file 41467_2022_31305_MOESM3_ESM.pdf]

Title: Supplementary Dataset 1.
Description: Strain origins and functional annotation of PGNC open reading frames.

Title: Supplementary Dataset 2.
Description: Full sequence and annotation of the circular PGNC in Genbank format.

Title: Supplementary Dataset 3.
Description: BioLog Phenotype Microarray data.
